# Unlocking the genome of the non-sourdough *Kazachstania humilis* MAW1: insights into inhibitory factors and phenotypic properties

**DOI:** 10.1186/s12934-024-02380-7

**Published:** 2024-04-15

**Authors:** Damian Mielecki, Anna Detman, Tamara Aleksandrzak-Piekarczyk, Małgorzata Widomska, Aleksandra Chojnacka, Anna Stachurska-Skrodzka, Paulina Walczak, Elżbieta Grzesiuk, Anna Sikora

**Affiliations:** 1 Institute of Biochemistry and Biophysics, Polish Academy of Sciences, Pawińskiego 5a, Warsaw, 02-106 Poland; 2 Present Address: Mossakowski Medical Research Institute, Polish Academy of Sciences, Pawińskiego 5, Warsaw, 02-106 Poland; 3 Institute of Biology, Warsaw University of Life Sciences, Nowoursynowska 159, Warsaw, 02-776 Poland; 4 Centre of Postgraduate Medical Education, Marymoncka 99/103, Warsaw, 01-813 Poland

**Keywords:** Ascomycetous budding yeasts, *Kazachstania humilis*, Genome sequencing, Genome assembly, Inhibition of bacterial growth, Fermentation, Next generation sequencing, Oxford Nanopore, Illumina

## Abstract

**Background:**

Ascomycetous budding yeasts are ubiquitous environmental microorganisms important in food production and medicine. Due to recent intensive genomic research, the taxonomy of yeast is becoming more organized based on the identification of monophyletic taxa. This includes genera important to humans, such as *Kazachstania*. Until now, *Kazachstania humilis* (previously *Candida humilis*) was regarded as a sourdough-specific yeast. In addition, any antibacterial activity has not been associated with this species.

**Results:**

Previously, we isolated a yeast strain that impaired bio-hydrogen production in a dark fermentation bioreactor and inhibited the growth of Gram-positive and Gram-negative bacteria. Here, using next generation sequencing technologies, we sequenced the genome of this strain named *K. humilis* MAW1. This is the first genome of a *K. humilis* isolate not originating from a fermented food. We used novel phylogenetic approach employing the 18 S-ITS-D1-D2 region to show the placement of the *K. humilis* MAW1 among other members of the *Kazachstania* genus. This strain was examined by global phenotypic profiling, including carbon sources utilized and the influence of stress conditions on growth. Using the well-recognized bacterial model *Escherichia coli* AB1157, we show that *K. humilis* MAW1 cultivated in an acidic medium inhibits bacterial growth by the disturbance of cell division, manifested by filament formation. To gain a greater understanding of the inhibitory effect of *K. humilis* MAW1, we selected 23 yeast proteins with recognized toxic activity against bacteria and used them for Blast searches of the *K. humilis* MAW1 genome assembly. The resulting panel of genes present in the *K. humilis* MAW1 genome included those encoding the 1,3-β-glucan glycosidase and the 1,3-β-glucan synthesis inhibitor that might disturb the bacterial cell envelope structures.

**Conclusions:**

We characterized a non-sourdough-derived strain of *K. humilis*, including its genome sequence and physiological aspects. The MAW1, together with other *K. humilis* strains, shows the new organization of the mating-type locus. The revealed here pH-dependent ability to inhibit bacterial growth has not been previously recognized in this species. Our study contributes to the building of genome sequence-based classification systems; better understanding of *K.humilis* as a cell factory in fermentation processes and exploring bacteria-yeast interactions in microbial communities.

**Supplementary Information:**

The online version contains supplementary material available at 10.1186/s12934-024-02380-7.

## Background

Yeasts are unicellular eukaryotic microorganisms in the kingdom *Fungi* that form a polyphyletic group. In recent years, the taxonomy of yeasts has changed as the development of sequencing technologies resulted in an increasing number of completed genomes, which form the basis of genome sequence-based classification and identification systems [1–4]. A good example of the reassignment and reclassification of yeast species are the changes within the genera *Candida*, *Saccharomyces*, and *Kazachstania*, belonging to the phylum *Ascomycota* [5–7],. The genus *Kazachstania* (40 species recognized to date) is most closely related to the genera *Naumovozyma* and *Saccharomyces*, which also fall within the family *Saccharomycetaceae* (https://eol.org/pages/6655123/names). Furthermore, studies on the evolution of genomes have helped to distinguish clades, i.e. monophyletic groups of organisms possessing a common ancestor, and the term *Kazachstania* clade is now commonly used [5, 8].

*Kazachstania* species have been isolated from a wide variety of habitats in different areas of the globe, mainly from the soil, e.g. *Kazachstania solicola* [9], *Kazachstania taianensis* [10], and *Kazachstania telluris* [11]; but also from naturally fermented foods, e.g. *Kazachstania humilis* [12], *Kazachstania saulgeensis* [5], *Kazachstania bulderi* [13]; plants, e.g. *Kazachstania rupicola* [14]; silage, e.g. *Kazachstania aerobia* [15] rotting wood, e.g. *Kazachstania serrabonitensis* [5]; spoiled food, e.g. *Kazachstania hellenica* [16]; wastewater, e.g. *Kazachstania aquatica* [9]; and even from animals, e.g. *Kazachstania slooffiae* [11] and faeces, e.g. *Kazachstania heterogenica* [11]. After *Saccharomyces cerevisiae*, *Kazachstania humilis* is the yeast found most frequently in sourdoughs and other naturally fermented foods. This yeast was named “Microbe of the Month” [17] after the sequenced genome of a strain isolated from agave fermentation was published in October 2022 [18].

Previously, we isolated ascomycetous budding yeasts related to *Kazachstania humilis* (isolate MAW1), *K. exiqua* and *Geotrichum candidum* from dark fermentation bioreactors processing by-products of the sugar industry, and identified them as inhibitors of bio-hydrogen production [19]. These yeasts caused instability of the microbial communities in these reactors due to a metabolic shift, quantitative changes in the fermentation products, a decrease in pH, and inhibition of bacterial growth. The primary source of these yeasts was most probably sugar beet [19]. Using a panel of Gram-positive [*Bacillus megaterium, Clostridium butyricum*, various lactic acid bacteria (LAB)] and Gram-negative (*Escherichia coli, Pseudomonas putida, Citrobacter freundii, Klebsiella oxytoca*) indicator bacteria we demonstrated the secreted yeast metabolites produced in the acidic environment inhibit bacterial growth under both aerobic and anaerobic conditions [19]. Since the *K. humilis* isolate MAW1 produced the greatest inhibitory effect, this strain was selected as the object of the present study.

The antagonistic activity of yeasts against other microorganisms is at the forefront of the search for novel antimicrobial compounds. Notably, yeasts also show antiviral activity. The anti-fungal and anti-bacterial activities of yeasts include competition for nutrients, pH changes caused by their metabolic activity, and the production of metabolites such as ethanol, volatile acids, hydrogen peroxide, etc. Furthermore, yeasts produce killer toxins (mycocins). The mechanisms of mycocin action are most well characterized for anti-fungal (including other yeasts) agents and can be divided into several categories: (i) extracellular proteins that hydrolyze β-1,3-D-glucans in fungal cell walls or inhibit their synthesis leading to cell wall damage; (ii) proteins causing the disruption of cell membranes which promotes ion leakage; (iii) proteins blocking DNA synthesis and cell division; (iv) tRNA-cleaving nucleases; (v) polypeptides blocking calcium uptake; and (vi) proteins promoting rRNA fragmentation [20–23]. Similar, less well-studied, mechanisms have been confirmed for antibacterial factors, i.e. disintegration of bacterial cell envelopes, increased membrane permeability, and inhibition of cell growth. Short peptides and small proteins, extracellular biosurfactants known as sophorolipids, and secondary metabolites (e.g. phenyllactic and indollactic acids) have been identified as among the factors responsible [20, 21, 24–28]. To date, studies on the antibacterial properties of yeasts have mainly focused on pathogenic bacteria and those involved in food spoilage. Hipp and coworkers (1974) were the first to show that the medium in which *Candida albicans* had been cultured could inhibit the growth of several *Neisseria gonorrhoea* strains [29]. Furthermore, several yeast species including *Kluyveromyces marxianus* and *Geotrichum candidum* display considerable anti-listerial activity [26, 30]. Detman and colleagues (2018) identified β-1,3-glucosidase, which might disturb the structures of both the bacterial cell envelope and biofilms, as a potential factor inhibiting bacterial growth produced by yeasts [19].

Up to 2021, among 103 yeast strains from 46 genera that were examined, only 8.7% displayed any antimicrobial activity [20, 22]. Within the genus *Kazachstania*, such killer activity was detected in strains of *K. exigua* [31, 32], *K. lodderae* [33] and *K. unispora* [31] (for a review see [22]). Notably, all of these species have been reclassified from other genera including *Candida*, *Saccharomyces* and *Kluyveromyces*.

Interestingly, crude filtrates obtained after culturing *K. humilis* MAW1 in a neutral (pH 7.0) medium were found to stimulate the growth of some bacteria [19]. This supports the notion that yeasts are a source of nutritive compounds such as vitamins, amino acids, organic acids, and other metabolites that support bacterial growth, which is the reason why yeast extract is frequently included in culture media [21, 34, 35].

In this study we characterize *K. humilis* isolate MAW1 by sequencing and analysing its genome. The genome was sequenced in diploid state allowing the further research, including SNPs (single nucleotide polymorphisms) and INDELs (insertion and deletions). The mating-type (MAT) locus is organized distinctly as compared to other species of *Kazachstania*. The global phenotypic profiling of the *K. humilis* MAW1 was provided. The species demonstrated the inhibitory activity on bacterial growth (on the model strain *E. coli* AB1157), and by demonstrating its activities that inhibit cell division by filament formation in a pH-dependent manner. This is the first in-depth analysis of a non-sourdough-derived isolate of *K. humilis* and the first report of inhibitory activity against bacteria in this species. Our study provides new knowledge and reveals new properties of the species regarded as the second most commonly identified sourdough yeast after *S. cerevisiae*.

## Methods

### Microbial strains and growth media

*K. humilis* MAW1 was isolated from dark fermentation bioreactors processing by-products of the sugar beet industry [19]. The tester bacterial strain used was *Escherichia coli* K12 AB1157 (*argE3, hisG4, leuB6*, Δ*(gpt -proA)62, thr-1, ara-1, galK2, lacY1, mtl-1, xylA5, thi-1, rpsL31, glnV44, tsx-33, rfbD1, mgl-51, kdgK51*) [36]. This strain is frequently employed in studies on bacterial responses to a variety of mutagenic agents [37]. The growth media used were lysogeny broth (LB; 1% Bacto-tryptone, 0.5% yeast extract, 0.5% NaCl) [38], C salts (0.02% MgSO_4_ × 7H_2_O, 1% K_2_HPO_4_, 0.2% citric acid, 0.35% NH_4_NaHPO_4_ × 4H_2_O) [39] and YPD (1% Bacto yeast extract, 2% Bacto peptone, 2% dextrose) [40]. As required, YPD medium was buffered to pH 4.5 using 0.1 M citrate-phosphate buffer [27]. For plates, LB or E-arg [C salts plus thiamine, glucose and a mixture of amino acids (proline, leucine, threonine, histidine), each at 25 µg/ml] were solidified with 1.5% Difco agar.

### Effect of *K. humilis* MAW1 on the growth of *E. coli *AB1157

*K. humilis* MAW1 was grown aerobically in YPD medium (pH 4.5 or 7.0) at room temperature (22–24 °C) without shaking for 72 h and then the cultures were filtered through a 0.2-µm PES membrane using a VWR Vacuum Filtration System. The crude filtrates were mixed with an equal volume of LB medium and 3 ml lots were inoculated with 20 µl of an overnight culture of *E. coli* AB1157 grown in LB. Control cultures were prepared using YPD (pH 4.5 or 7.0) instead of the crude filtrate or just LB medium. The mixtures of LB: acidic YPD and LB: yeast filtrate after cultivation in acidic conditions had a pH of ∼ 5.0. After overnight incubation at 37 °C with shaking, the bacterial cultures were (i) diluted in C salts, plated on LB plates, incubated overnight at 37 °C and the colonies counted to determine the number of viable cells/ml, and (ii) plated on E-arg plates and incubated for two days at 37 °C to evaluate the frequency of Arg^+^ revertants (per 10^7^ cells). The resulting survival rates were compared with two sided t.test function and graph prepared in ggplot2 3.4.0 and ggsignif 0.6.4 packages [41, 42], in R 4.2.2 project. The statistical significance of t.test was set according to p-value as follows, 0.05 ≥ * > 0.01 ≥ ** > 0.001 ≥ ***. The bacteria were also stained with basic fuchsin (Sigma-Aldrich) and examined under a light microscope (Nikon Microphot-SA) with a 100× objective lens. Cells were photographed at 1000-fold magnification. The microscopic images were processed in Fiji 2.11.0, mainly by Enhance Local Contrast (CLAHE) and Subtract Background functions [43] for better visualization.

### Phenotypic profiling using phenotype MicroArrays

Phenotype MicroArray (PM) MicroPlates FF, PM9 and PM10 (Biolog Inc., USA) were applied to test the ability of *K. humilis* MAW1 to utilize 95 different carbon sources (amides, amines, amino acids, carboxylic acids, polyalcohols, esters, glycosides, nucleosides, phosphates, polymers and saccharides), and also examine its osmotic sensitivity, resistance to toxic ions and pH tolerance. Quantitation of phenotypes with the PM assay was performed using Biolog’s patented redox technology, with cell respiration or fermentation (via NADH production) as a universal reporter. Metabolic activity causes the reduction of a tetrazolium dye to produce a strong colour in the plate wells. Fresh colonies of *K. humilis* MAW1 were suspended in FF inoculation fluid deficient in carbon sources (Biolog Inc., USA) to a final transmittance of 70% and 100 µl lots were transferred to the wells of the FF MicroPlate. For the PM9 and PM10 panels, a 62% T (transmittance) cell suspension was diluted 1:48 in SC medium [SC Amino Acid Mixture (MP Biomedicals, Germany), Yeast Nitrogen Base (Difco, France), 100 mM glucose, plus 100-fold diluted E dye (Biolog Inc., USA)] and this was used to inoculate the relevant PM MicroPlate wells as described above. All plates were incubated aerobically in an Omnilog incubator-plate reader (Biolog Inc., USA) for 72 h at 30 °C. Metabolic activity was monitored kinetically by colorimetric measurement every 15 min. Preliminary data analysis was performed using the Biolog Kinetic and Parametric software (Biolog Inc., USA). The data represent average values of the area under the curve (AUC). The assay was repeated using at least two MicroPlate.

### Isolation of genomic DNA

Genomic DNA (gDNA) was isolated from an overnight culture of *K. humilis* MAW1 grown in 5 ml of YPD medium at 30 °C. The yeast cells were harvested by centrifugation (10,000 × g, 2 min), resuspended in 10 ml of lysis buffer (50 mM potassium phosphate pH 7.5, 1 M sorbitol, 5 mM EDTA, 10 U zymolyase, 0.4 mg of RNAse A) and incubated at 37 °C for 1 h. The resulting spheroplasts were collected by centrifugation (10,000 × g, 5 min) and resuspended in CTAB buffer (2% cetyl trimethylammonium bromide, 100 mM Tris-HCl pH 8.0, 1.4 M NaCl, 20 mM EDTA) containing 0.1 mg/ml of proteinase K. After cell lysis was completed by incubation at 60 °C for 1 h, cell debris was pelleted by centrifugation (15,000 × g, 10 min), and gDNA was extracted from the supernatant using phenol-chloroform-isoamyl alcohol [PCI; 25:24:1; prepared using phenol saturated with 10 mM Tris-HCl pH 8.0, 1 mM EDTA (Sigma-Aldrich)]. An equal volume of PCI was added to the supernatant and the phases were mixed by inversion. After incubation at room temperature for 5 min the mixture was centrifuged (10,000 × g) for 10 min. The aqueous phase was transferred to a new tube and the extraction with PCI repeated. The aqueous phase was then extracted three times with an equal volume of chloroform-isoamyl alcohol (24:1). The gDNA was precipitated with 0.7 volumes of isopropanol, pelleted by centrifugation, washed with 70% ethanol and air-dried before resuspending in sterile milliQ water. The concentration and purity of the gDNA was assessed using a NanoDrop spectrophotometer. The integrity of the gDNA was checked using pulsed-field gel electrophoresis, which showed that the preparation was comprised of DNA fragments of ≥ 20 kbp in length.

### Sequencing of *K. humilis* MAW1 gDNA

Sequencing of the *K. humilis* MAW1 genome was performed using a 2 × 300 PE MiSeq strategy in the DNA Sequencing and Synthesis Facility at IBB PAS. For Oxford Nanopore MinION sequencing, gDNA of *K. humilis* MAW1 was fragmented into 8–10 kbp fragments using a Covaris gTUBE (7200 rpm for 60 s). The gDNA was then processed with a Ligation Sequencing Kit 1D (Oxford Nanopore, SQK-LSK108). Sequencing was performed with a R9.4 flow cell (Oxford Nanopore, FLO-MIN106) in a MinION Mk1B.

### Filtering of raw sequencing reads

The quality of raw reads was assessed with FASTQC 0.11.8 for Illumina reads or NanoStat 1.1.2 for Nanopore reads [44]. The Illumina reads were trimmed to remove adapter sequences (TruSeq3_IndexedAdapter: AGATCGGAAGAGCACACGTCTGAACTCCAGTCAC, TruSeq3_UniversalAdapter: AGATCGGAAGAGCGTCGTGTAGGGAAAGAGTGTA) and at the ends of reads according to the quality score (< 30 Phred score) using Trimmomatic 0.33 [45] with the parameters ILLUMINACLIP: TruSeq3-SE.fa:2:30:10 LEADING:30 TRAILING:30 SLIDINGWINDOW:4:20 MINLEN:70.

The Nanopore fasta5 files were transformed to fastq with Poretools 0.6.0 [46], and filtered using the minimum Phred score of 10 and minimal length of 500 nt with NanoFilt 2.0.1 [44].

### *K. humilis* MAW1 genome length prediction

The Illumina reads were used to predict the genome size of *K. humilis* MAW1. The frequency of 21-kmers was assessed with Jellyfish 2.0 [47] using the following parameters: count -t 1 -C -m 21 -s 1G. The data were visualized with GenomeScope [48].

### Genome assembly of *K. humilis* MAW1

Short Illumina and long Nanopore reads were combined in the assembly of the *K. humilis* MAW1 genome. Two approaches were employed: (i) Sequence assembly with long reads and polishing with short reads. For the assembly of long reads Canu 1.8 [49] was used. Subsequently, the short reads were mapped to the contigs with BWA MEM 0.7.17 [50] with default parameters, sorted with SAMtools [51], and error corrected with Pilon 1.22 [52], using the parameters --fix all --mindepth 0.5 --changes –verbose; (ii) Sequence assembly with short paired reads and hybrid assembly performed with SPAdes 3.11.1 [53], using default parameters. The outcome assemblies were verified with QUAST 5.0.2 [54], particularly the number of contigs and their total length, N50, L50, GC content, and single copy orthologues. The following parameters were used: --fungus --conserved-genes-finding.

For the calculation of assembly coverage and visualization of deletion/duplication regions, the Illumina reads were filtered, corrected, and the first base of every read removed with fastp, retaining 91.96% [55]. Reads were mapped to the *K. humilis* MAW1 assembly with bwa mem [56]. The coverage depth was calculated with samtools depth tool [51], processed by moving median (window: 1000) with RcppRoll R package. Additionally, the telomere repeats were searched with the tdik tool with default parameters [57]. All data were visualized with ggplot2 [41], and all procedures conducted within R 4.2.2 project [58].

### Prediction of ORFs/genes in the *K. humilis* MAW1 genome assembly

BRAKER 2.1.2 was used for gene prediction [59, 60], with the following parameters: --esmode –fungus. The rRNA genes were identified with RNAmmer 1.2 server [61], and tRNA genes with tRNAscan-SE 2.0 server [62].

A custom python script was used for the distribution of gene length. The following assemblies of other yeast genomes were selected for comparison: *Candida albicans* SC5314 (TaxID: 237,561, Assembly: ASM18296v3), *Nakaseomyces glabratus* CBS 138 (TaxID: 5478, Assembly: ASM254v2), *Candida tropicalis* MYA-3404 (TaxID: 294,747, Assembly: ASM633v3) (Butler et al., 2009), and *Lodderomyces elongisporus* NRRL YB4239 (TaxID: 379,508, Assembly: ASM14968v1) (NCBI Database).

### Ploidy assessment and MAT locus analysis

The ploidy assessment of *K. humilis* MAW1 was performed by the flow cytometry and of MAT locus analysis as well as by the allele frequency determination. The cytometry analysis of the *K. humilis* MAW1 cells was performed mainly according to [63]. The *Saccharomyces cerevisiae* BY4741 MATa *his leu ura met* (haploid) and *S. cerevisiae* BY4743 (diploid) were used as control strains. Briefly, the overnight cultures were used to inoculate fresh YPD medium and grown to OD_600_ = 0.6. Subsequently, yeast cells were centrifuged (500 × g, 3 min), resuspended rapidly in 2 volumes of 70% ethanol, and stored overnight at 4 °C, centrifuged (500 × g, 3 min), resuspended in 1 volume of 50 mM sodium citrate, and the step was repeated. Cells were sonicated for 5 s to ensure that cells were dispersed in solution, eliminating cell clumps, centrifuged (500 × g, 3 min), resuspended in 0.4 volumes of 50 mM sodium citrate supplemented with 0.5 mg/ml RNase A, and incubated for 2 h at 37 °C with occasional inversion. Fifteen µl aliquot of unstained cells was used as a negative stain control. A volume 185 µl of fixed cells were stained overnight in the dark with PI (25 µg/ml) at 37 °C. Immediately before analyzing on the flow cytometer, samples were homogenized by sonication. Stained cells underwent a FACS analysis using FACSCanto II flow cytometer and FACSDiva software (Becton Dickinson, USA). Flow rate was about 1000 events/cells per second. Signals from 20.000 events per sample were captured. Gating strategy: forward scatter-A (FSC-A) and side scatter -A (SSC-A) gate was set to exclude cell debris, and FSC-A/ FSC-H gate was set to exclude cell doublets. Ploidy was estimated on the basis of mean fluorescence intensity of PI on a linear scale.

In the case of the MAT locus analysis, tblastn searches [64] were performed with the MAT locus genes and their orthologues from *Saccharomyces cerevisiae* S288C, being MATALPHA1 (locus tag: YCR040W), MATALPHA2 (YCR039C), HMRA1 (YCR097W), and HMRA2 (YCR096C), from *Kazachstania naganishii* CBS8797, being MATALPHA1 (KNAG_0C00150), MATALPHA2 (KNAG_0C00160), and MATA1 (KNAG_0C00795), and *Kazachstania africana* CBS 2517, being MATALPHA1 (KAFR_0D00710), MATALPHA2 (KAFR_0D00720), and MATA1 (KAFR_0G00180). Additionally, the homologues of genes flanking MATA1, MATALPHA1, or MATALPHA2 in K. *humilis* YMX004033 and *K. humilis* MAW1 assemblies, as well as all main genes connected with mating were searched with tblastn [64]. All the gene analysis and graphing was performed in Geneious 10.2.6 [65]. Bcftools mpileup, call, and norm were used to produce INDELs and SNPs information, based on mapping produced for coverage calculation [66]. The custom bash script was used to retrieve selected columns of vcf file, and data were prepared in R [58], by filtering INDELs and SNPs by quality > 200, and calculating allele frequencies. Graphs were prepared with ggplot2 R package [41].

### Phylogenetic classification of *K. humilis* MAW1

The preliminary blastn searches with the *K. humilis* MAW1 18 S rDNA sequences against NCBI nr database pointed towards *K. humilis* species [64, 69]. The databases consisting of 18 S rDNA, ITS-D1-D2 cluster, or 18 S-ITS-D1-D2 cluster were prepared by downloading mainly the reference genomes for *Saccharomycetaceae* (taxid 4893) from NCBI database. These databases were mapped with the primer pairs NS1/NS8, ITS1/NL-4, and NS1/Nl-4 [70] and *in silico* PCR products were produced. If one assembly/genome produced more than one PCR product, the sequences were aligned with ClustalW (cost matrix: IUB, gap open cost: 15, gap extend cost: 6.66) [71], and the consensus sequence was produced for > 90% identity alignment. If the alignment produced presented < 90%, the sequences were grouped according to similarities, and the algorithm was run once again until the consensus could be retrieved. Subsequently, the sequences in three databases were aligned with ClustalW with the same parameters, and phylogenetic threes were produces with Tamura-Nei genetic distance model [67], UPGMA (unweighted pair group method with arithmetic mean) method [68], and bootstrapping with 1000 replicates, all peformed in Geneious 10.2.6 [65]. Only the trees based on the alignment performed with the 18 S-ITS-D1-D2 cluster are shown.

### Identification of genes with putative toxic activity against bacteria

To identify genes with putative antibacterial activity in the *K. humilis* MAW1 genome assembly, proteins with established antimicrobial activity, as well as those accompanying the so-called killer activity of yeasts, were selected based on literature searches. These are presented with information retrieved from UniProtKB [72] and selected publications in Supplementary Tables 1 and 2 [see Additional file 1]. Only proteins for which the sequence information was deposited in either the UniProtKB or NCBI databases were chosen. The UniProtKB IDs were used as sequence identifiers. To search the *K. humilis* MAW1 assembly, tblastn was used with the following parameters: matrix – BLOSUM62, Gap cost – 11, Gap extend – 1, Max E-value – 10, Word size – 6 [64]. All results are presented in Additional files 2 and 3. Significant results, with an E-value of < ∼ 1 and/or Query coverage > ∼ 50%, are presented in the main text.

### Database entries

The Illumina and Nanopore sequencing results were uploaded under the NCBI BioProject PRJNA785806 and two BioSamples: SAMN23578054 (for Illumina reads) and SAMN23578055 (for Nanopore reads).

## Results

### Assembly and annotation overview of the K. Humilis MAW1 genome

After filtering the raw Illumina data, 935,589 reads remained, with an average Phred score of 29.6. These accounted for 212 and 167 Mbases (84.62%), for forward and reverse reads, respectively [see Supplementary Table 3 in Additional file 1]. After filtering the Nanopore reads based on a quality score of 11, 291,705 (75%) remained, with an average length of 8188 bases per read and representing over 2.3 Gbases in total [see Supplementary Table 4 in Additional file 1].

**Fig. 1:**
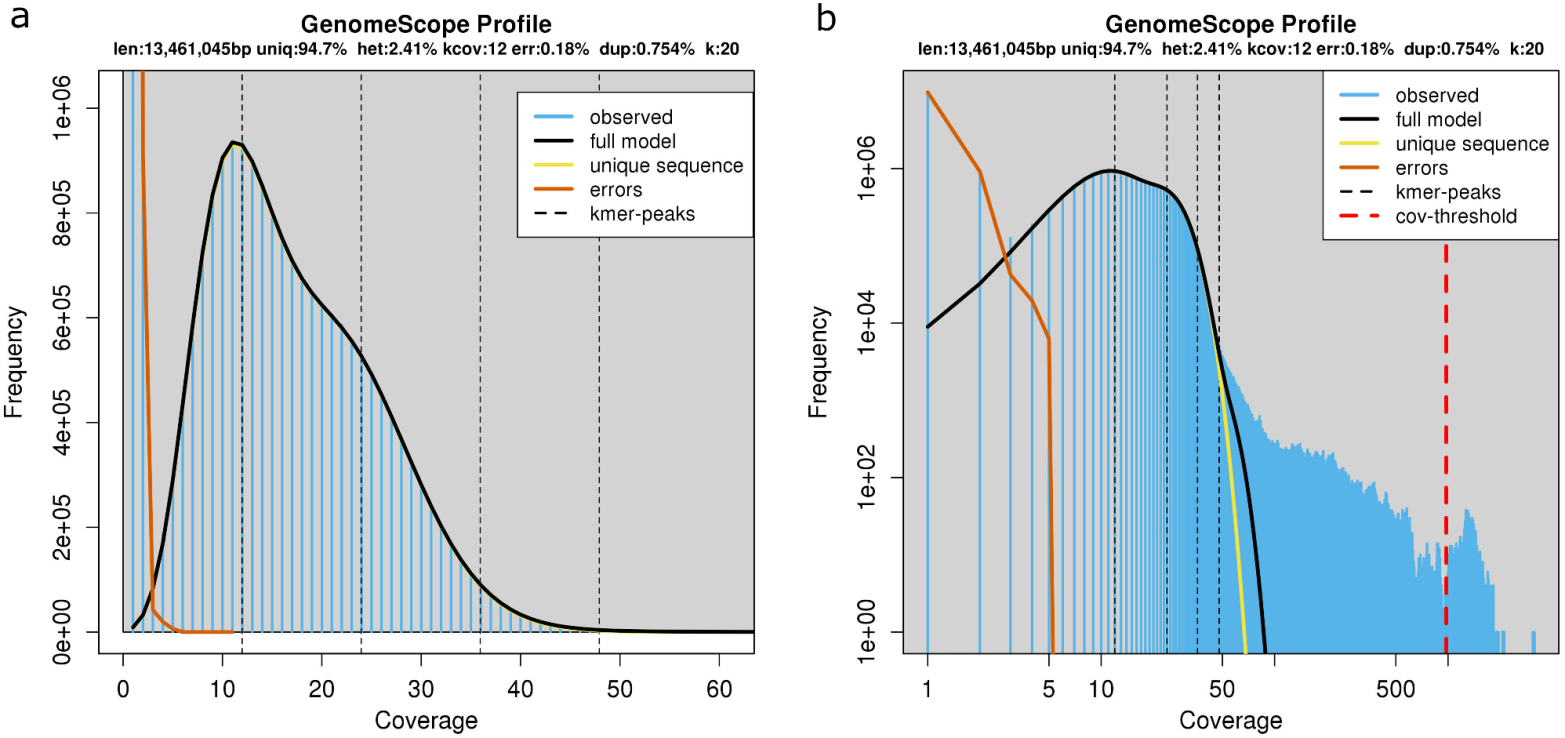
K-mers distribution of Illumina reads (**A** – regular scale, **B** – log scale)

The GenomeScope profile indicated that the probable size of the haploid *K. humilis* MAW1 genome is 13.46 Mbp. Repeated sequences comprised 0.3% and heterozygosity was 2.38%. Reads showed a 0.179% error level. The model fit was 99.47% (Fig. 1).

Different strategies used for the assembly of reads gave substantially different results (Table 1). The least number of contigs, 57 sequences, was obtained with Canu. Errors in the sequences were corrected by short reads with Pilon. This strategy resulted in an N50 of 1,118,589, with 5 sequences. This assembly permitted identification of 94.83% of complete single-copy orthologous genes. On the other hand, the hybrid assembly conducted with SPAdes resulted in as many as 3416 contigs, with an N50 of 28,419, containing 188 sequences. As expected, the assembly based only on short reads gave the greatest number of contigs, 9297, with an N50 of 3765 (with 1349 sequences). Using the Canu strategy, contigs with a total length of 15.42 Mbases were obtained. In comparison, the two SPAdes strategies resulted in totals of 21.5 to 23.0 Mbases. Thus, the Canu strategy gave an assembled contigs size much closer to the predicted genome size of *K. humilis* MAW1, namely 13.46 Mbases. This genome size is similar to those of other yeast species, e.g. 14.7 Mbases for *C. albicans* or 12.3 Mbases for *C. glabrata* (NCBI, 2019). Therefore, the Canu/Pilon assembly was used for all subsequent analysis.

**Table 1:** Statistics of *K. humilis* MAW1 genome assembly strategies used in the study (Canu, only Nanopore reads; Canu/Pilon, assembly of Nanopore reads polished with short reads; SPAdes-hybrid, both long and short reads; SPAdes-PE, only Illumina reads)

Parameter	Canu	Canu + Pilon	SPAdes-hybrid	SPAdes-PE
# contigs	57	57	3416	9297
Largest contig	2,297,614	2,307,406	565,621	216,146
Total length	15,353,986	15,422,215	23,043,389	21,499,633
GC (%)	48.87	48.94	48.98	49.04
N50	1,113,526	1,118,589	28,419	3765
N75	487,588	489,712	9119	1769
L50	5	5	188	1349
L75	9	9	567	3448
Complete BUSCO (%)	73.79	94.83	97.24	83.79
Partial BUSCO (%)	16.9	3.1	1.38	13.79

**Fig. 2:**
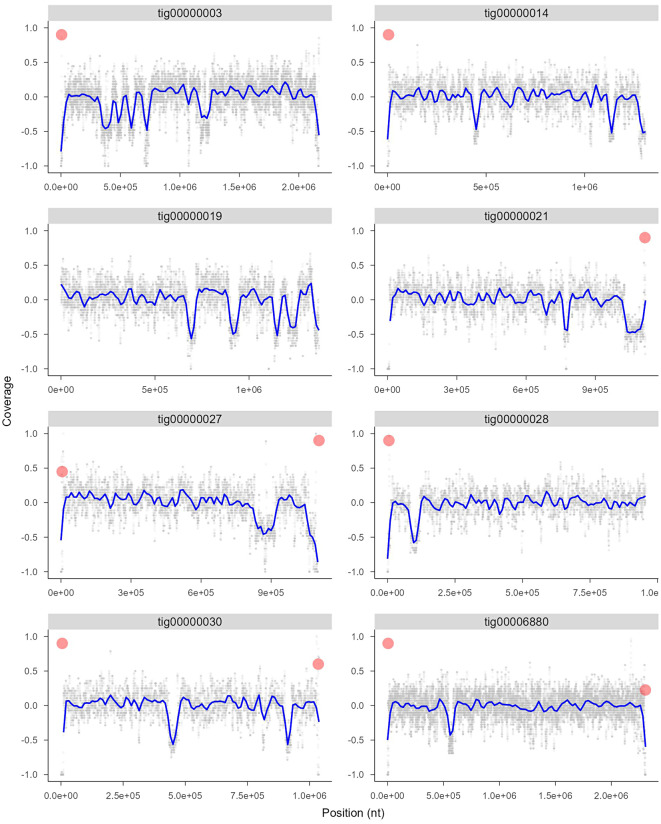
The assembly coverage for the eight longest contigs with the moving median (window 1000, every other 100 bp shown) showing the fragmental deletions in the diploid genome of *Kazachstania humilis* MAW1. Also the presence of the TCACGTGGAG repeat consisting of potential telomeres is shown (red dots)

The assembly obtained was further analysed for potential presence of duplication/deletion events and telomere regions. As show in Fig. 2 for the eight longest contigs, there is a prevalence of deletion of particular fragment, quite numerous in e.g. tig00000019. As shown by telomere repeats analysis (TCACGTGGAG sequence), most of the longest contigs is complete at least at one side and contigs tig00000027, tig00000030, and tig00006880 are telomere-to-telomere thus can be considered as full chromosomes (Fig. 2).

### Identification of genes in the *K. humilis* MAW1 assembly

Statistical analysis of the *K. humilis* MAW1 CDSs showed substantial similarity to the other selected yeast genomes (Table 2). BRAKER analysis identified 4856 genes in *K. humilis* MAW1, with numbers in other strains ranging from 5311 to 6258 (excluding the number of genes for *C. albicans* SC5314). The average gene length was 1726.03 bp, which is similar to that of other analysed strains:1454.48 to 2324.22. A blastx search [73], using the longest gene of *K. humilis* MAW1 as a query against the NCBI nr database [74] identified polypeptides with the highest similarity to the midasin MDN1 of *Kazachstania saulgensis*, *Kazachstania africana* CBS 2517, *Naumovozyma castellii* CBS 4309, *Naumovozyma dairenensis* and *S. cerevisiae*.

**Table 2:** Comparison of CDSs of *K. humilis* MAW1 with selected yeasts (gene sizes in base pairs)

Statistics	*C. albicans* SC5314	*C. glabrata* CBS 138	*C. tropicalis* MYA-3404	*L. elongisporus* NRLL YB4239	*K. humilis* MAW1
Number of genes	12,421	5311	6258	5802	4856
Shortest gene	90	105	153	153	204
Longest gene	15,108	14,643	11,427	10,491	14,505
Mean length	2270.75	2324.22	1454.48	2312.71	1726.03

### Ploidy and MAT locus of K. Humilis MAW1

**Fig. 3:**
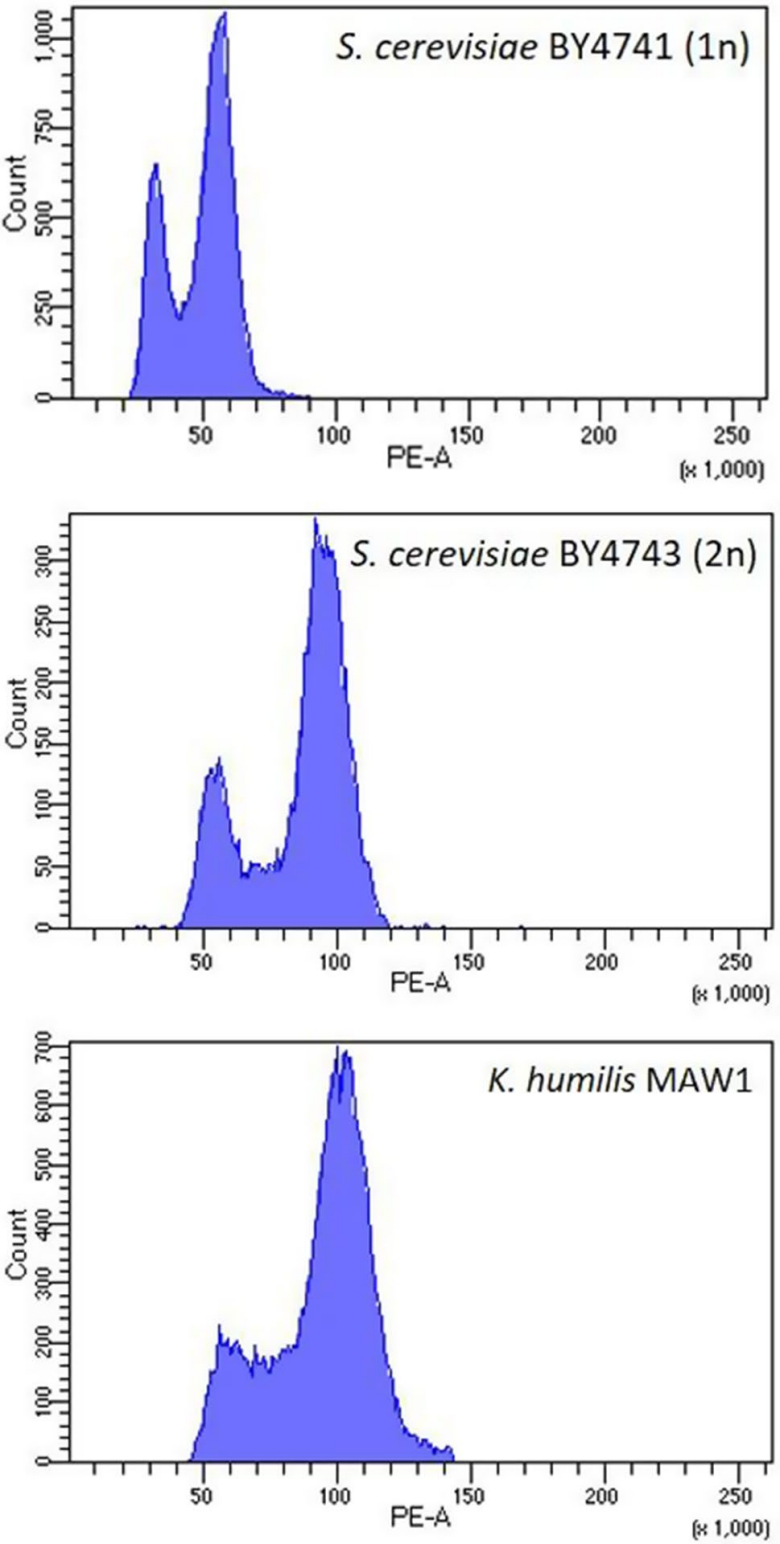
The propidium iodide flow cytometry analysis of two *Saccharomyces cerevisiae* BY4741 (haploid) and BY4743 (diploid) strains used as controls and the *Kazachstania humilis* MAW1 cells. The two peaks corresponding to cells in the haploid and diploid after mitosis states for *S. cerevisiae* BY4741, as well as diploid and tetraploid after mitosis states for *S. cerevisiae* BY4743 are visible. The almost perfect aligment of the peaks for *K. humilis* MAW1 with the *S. cerevisiae* BY4743 indicates that it exists in as diploid

**Fig. 4:**
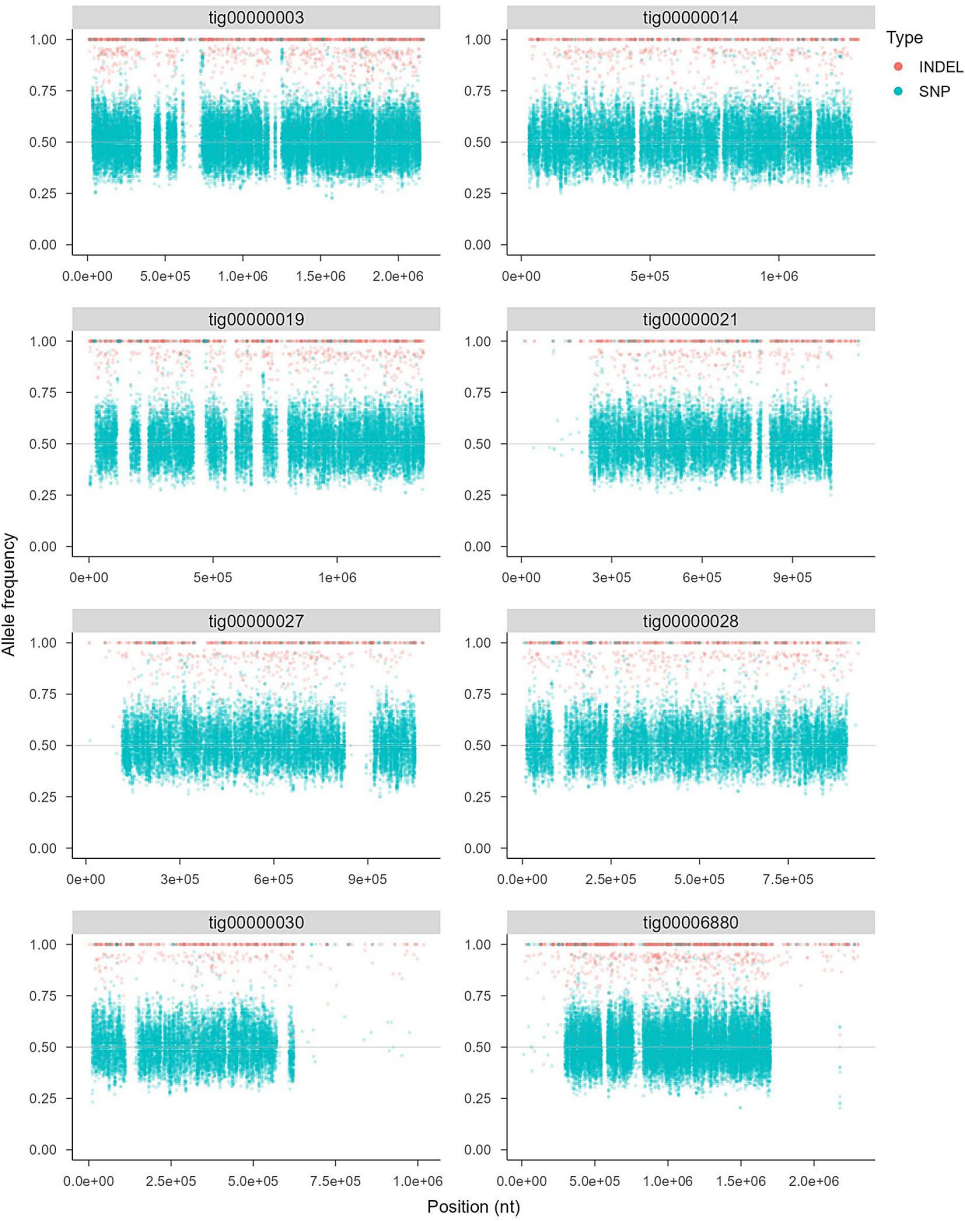
The allele frequency shown for the eight longest contigs of *K. humilis* MAW1 assembly. The frequencies are centered around 0.5 and 1 indicating the diploid state of the yeast

The genome size of the yeast was estimated to be 13.46 Mbp which is comparable to the genome size of *S. cerevisiae* S288C, 12.1 Mbp, making the *S cerevisiae* BY4741 (1n) and BY4743 (2n) strains a good control for the flow cytometry experiment. Since the two peaks of *K. humilis* MAW1 almost perfectly aligned with the *S. cerevisiae* BY4743, the genome was determined as to be diploid (Fig. 3). The allele frequency for the *K. humilis* MAW1 assembly is centred around 0.5 and 1 further corroborating the statement of its ploidy, as shown for the eight longest contigs (Fig. 4).

**Fig. 5:**
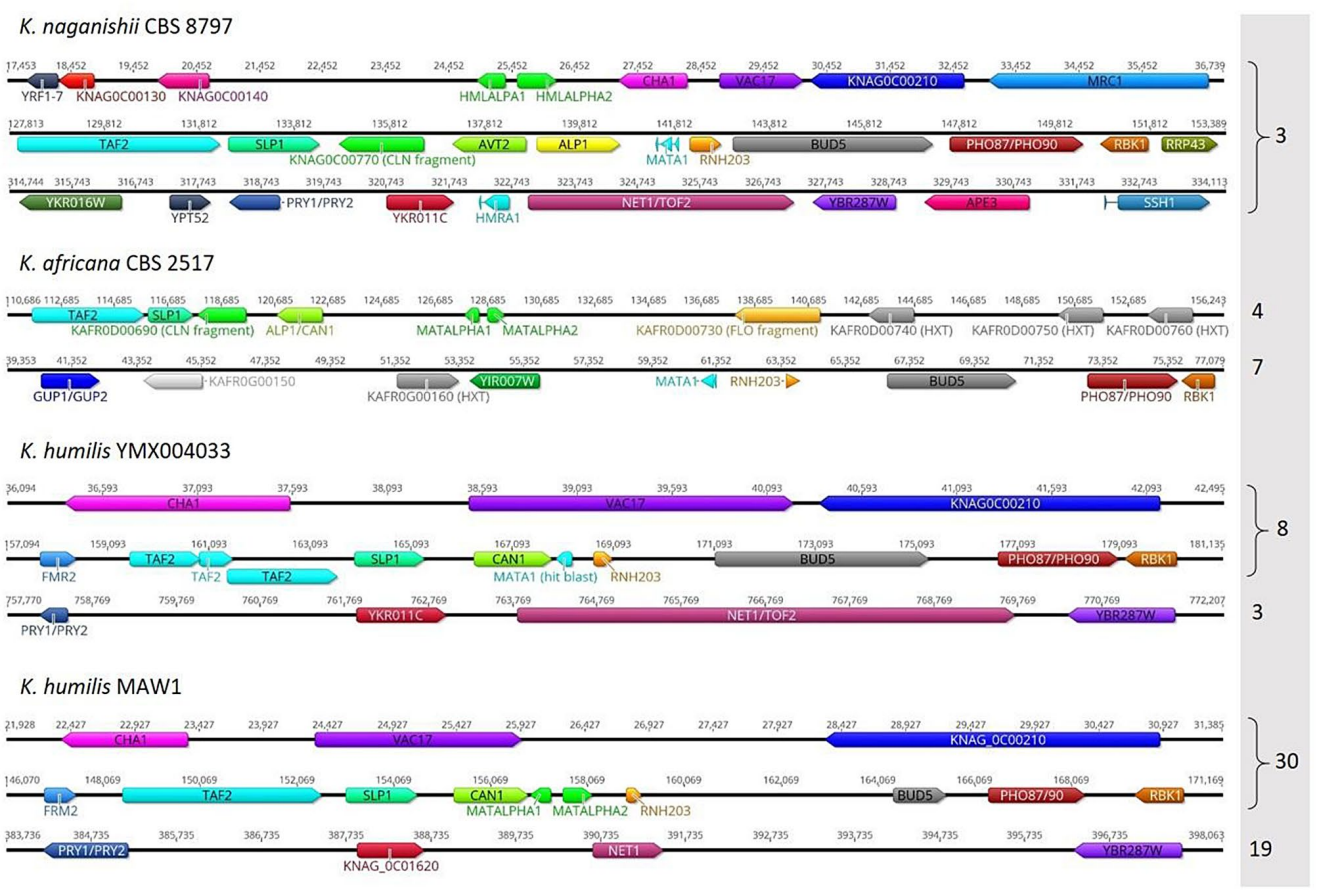
The genetic context of MAT loci for selected species, *Kazachstania naganishii* CBS 8797, *Kazachstania africana* CBS 2517, *Kazachstania humilis* YMX004033, and *Kazachstania humilis* MAW1. The gene homologues are shown in the same color to better visualize genetic rearrangements

As ploidy is inherently connected to the MAT locus, we dived into its comprehensive analysis. The *K. naganishii* CBS 8797, *K. africana* CBS 2517, and *K. humilis* YMX004033 were used as references (the *K. humilis* KAHU0 genetic context was identical to *K. humilis* YMX004033) The homologues of MATALPHA1 and MATALPHA2 were found residing in the contig tig00000030. The *K. naganishii* CBS8797 codes for MATA1 (being haploid) as well as both backup genes, HMLALPHA1, HMLAPLHA2 at the left arm of the same chromosome, and HMRA1 at the right arm (Fig. 5). The *K. africana* CBS 2517 genome probably underwent rearrangement losing both HML and HMR loci, and regaining genes for both MATA and MATALPHA type loci. Its MAT genes show only one side genetic context as it is in *K. naganishii* CBS 8797. The *K. humilis* strains (YMX004033, KAHU0, and MAW1) characterizes further rearrangement. In their genomes, only one MAT locus can be found (although MAW1 strain was diploid, the tblastn produced no results for MATA queries), either MATA1 or MATALPHA1/MATALPHA2, although its genetic context is the same as in *K. naganishii* CBS 8797. The arm of corresponding contigs (tig00000030 for MAW1 strain) shows the presence of genes right to the HML locus of this strain, indicating the loss of the rest of its sequence. On the other hand there is a contig (tig00000019 for MAW1 strain) that codes for genes surrounding the HMR locus of *K. naganishii* CBS 8797 but with no blast hit for MATA1 not MATA2 genes, the same in all three *K. humilis* strain analysed.

Moreover, the presence of other genes connected to mating was analysed in all three selected *K. humilis* assemblies. Each of them codes for at least two MFa as well as two MFα feromones, as well as STE2, FUS3, PTC1 (data not shown). Surprisingly, the three *K. humilis* YMX004033 and MAW1 strains do not produce HO recombinase, essential in mating type switching being similar to *K. africana* CBS 2517 but in opposite to *K. naganishii* CBS 8797 and *K. humilis* KAHU0. On the other hand, the IMEI methylase can be found in the MAW1 strains, thus making it differed from those two strains (data not shown). Nevertheless, the presented data need further research. The *K. humilis* MAW1 strain was also investigated for sporulation but no ascospores were observed during three weeks in *S. cerevisiae* sporulation medium (1% potassium acetate, 0.1% yeast extract, 0.05% glucose).

### Phylogenetic analysis of *K. Humilis* MAW1

**Fig. 6:**
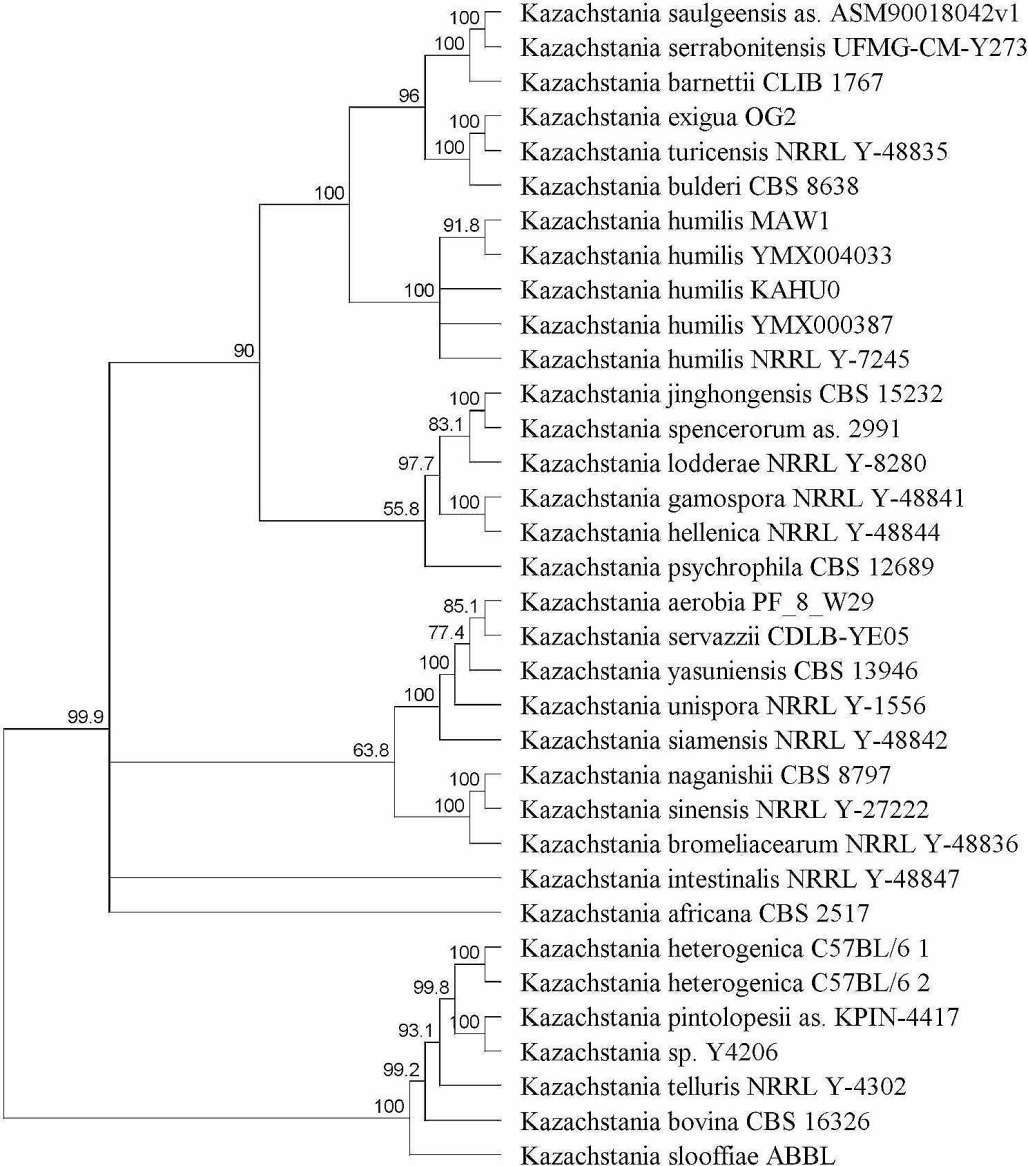
The UPGMA phylogenetic tree of the selected *Kazachstania* species based on the multiple alignment of 18 S-ITS-D1-D2 cluster, showing the clear distinction of *Kazachstania humilis* clade

The phylogenetic relationship of *K. humilis* MAW1 within *Saccharomycetaceae* family was elucidated on the bases of either 18 S–18 S-ITS-D1-D2 cluster. The strain clearly clustered with other *Kazachstania* species, especially with *K. humils* YMX004033 within the group consisting *Saccharomyces* genus (Additional file 4). Subsequently, the relationship within *Kazachstania* genus alone was investigated and shown that *K. humilis* MAW1 belongs to the cluster with other *K. humilis* species that are most closely related to *K. bulderi* CBS 8638 or *K. exigua* OG2. Quite surprisingly, *K. africana* CBS 2517 and *K. naganishii* CBS8797 belong to distinct clade (Fig. 6).

### Phenotypic profile of *K. humilis* MAW1

**Fig. 7:**
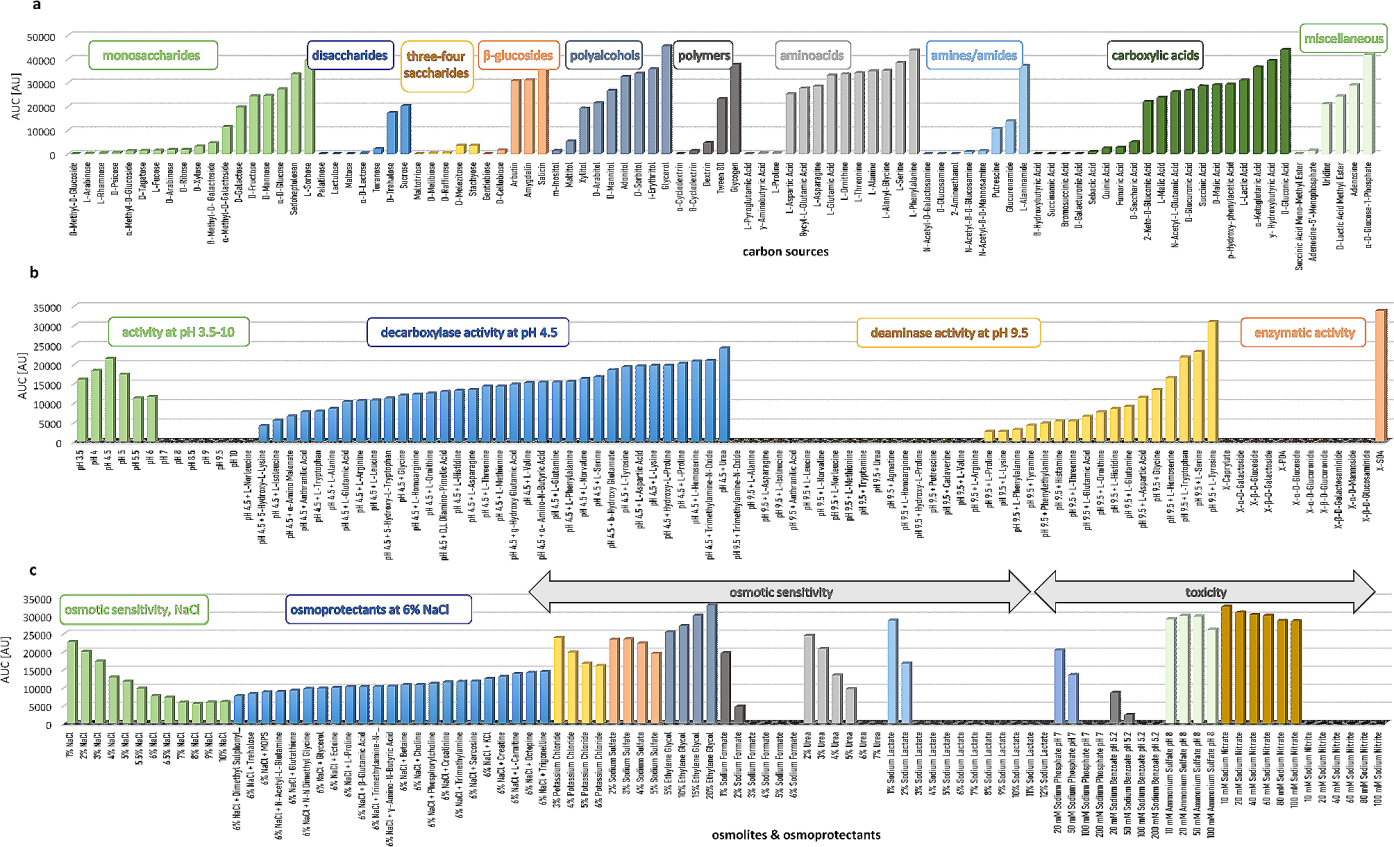
Phenotypic profile of *K. humilis* MAW1. Growth was monitored on a variety of carbon sources (**a**), over a range of pH values (**b**), and under different osmotic and ionic conditions (**c**)

The phenotypic profile of *K. humilis* MAW1 was determined using the FF, PM9 and PM10 MicroPlates (BIOLOG Phenotype MicroArrays) (Fig. 7). In total, 287 different growth conditions were tested, including 95 carbon sources, 96 osmotic and ionic conditions, and 96 pH environments.

As a carbon source, the isolate effectively utilized 7 out of the 18 monosaccharides tested (L-sorbose, sedoheptulosan, α-D-glucose and its derivative α-D-glucose-1-phosphate, D-mannose, D-fructose, D-galactose and α-methyl-D-galactoside), 2 out of 7 disaccharides (sucrose and D-trehalose), 3 out of 5 glycosides (salicin, amygdalin and arbutin), 7 out of 9 polyalcohols (glycerol, i-erythritol, D-sorbitol, adonitol, D-mannitol, D-arabitol and xylitol), 10 out of 13 amino acids (L-aspartic acid, glycyl-L-glutamic acid, L-asparagine, L-glutamic acid, L-ornithine, L-threonine, L-alanine, L-alanyl-glycine, L-serine and L-phenylalanine), 3 out of 8 amines/amides (L-alaninamide, glucuronamide and putrescine), 11 out of 19 carboxylic acids (D-gluconic acid, β-hydroxybutyric acid, α-ketoglutaric acid, L-lactic acid and its methyl ester, p-hydroxy-phenylacetic acid, D-malic acid, succinic acid, D-glucuronic acid, N-acetyl-L-glutamic acid, L-malic acid and 2-keto-D-gluconic acid) as well as adenosine and uridine.

The metabolic activity of *K. humilis* MAW1 under various stress conditions was tested using the PM9 and PM10 MicroPlates. In the PM9 MicroPlate test, this strain showed active metabolism in the presence of up to 10% sodium chloride, 6% potassium chloride, 5% sodium sulphate, 20% ethylene glycol, 2% sodium formate, 5% urea, or 2% sodium lactate. Of the toxic anions tested, MAW1 was resistant up to 50 mM sodium phosphate (pH 7.0), 50 mM sodium benzoate (pH 5.2), 100 mM ammonium sulphate (pH 8.0), 100 mM sodium nitrate, and was susceptible to all the tested concentrations of sodium nitrite (10–100 mM). In combinations of various osmoprotectants with 6% sodium chloride, *K. humilis* MAW1 performed well in all substrates, with the metabolic activity in most exceeding that in 6% NaCl alone. The PM10 MicroPlate test revealed that *K. humilis* MAW1 was most metabolically active at a pH of ≤ 6.0, with an optimal value of 4.5. It is noteworthy that *K. humilis* MAW1 grows well in YPD medium pH 7.0 in tube and flask tests, indicating the possible influence of the composition of the culture medium on the level of resistance to varying pH levels. Increased deaminase activity at pH 9.5 was observed in the tests with L-proline, L-lysine, L-phenylalanine, tyramine, phenylethylamine, histamine, threonine, L-glutamic acid, L-ornithine, L-histidine, L-glutamine, L-aspartic acid, glycine, L-homoserine, L-tryptophan, L-serine, and L-tyrosine. No metabolic activity was detected in the presence of X-caprylate, X-α-D-galactoside, X-β-D-glucoside, X-β-D-galactoside, X-PO_4_, X-α-D-glucoside, X-α-D-glucuronide, X-β-D-glucuronide, X-β-D-galactosaminide, X-α-D-mannoside, or X-β-D-glucosaminide, suggesting this strain’s failure to secrete enzymes that hydrolyse these substrates. The exception was efficiently metabolized X-SO4, indicating that *K. humilis* MAW1 produces an active sulphatase.

### Effects of *K. humilis* MAW1 on growth of *Escherichia coli *AB1157

Previously, we monitored the OD_600_ of different tester bacterial strain cultures to show the effects of adding crude *K. humilis* MAW1 culture filtrates [19]. Here, we focused on the survival of *E. coli* strain AB1157 by determining the number of viable cells. *E. coli* AB1157 was cultured under standard conditions (37 °C with shaking) in a 1:1 mixture of LB: filtrate of *K. humilis* MAW1 culture medium following growth in acidic (pH 4.5) or neutral (pH 7.0) YPD. *E. coli* AB1157 cultured in LB alone or 1:1 mixtures of LB: neutral YPD (pH 7.0) or LB: acidic YPD (pH 5.0) served as controls.

**Fig. 8:**
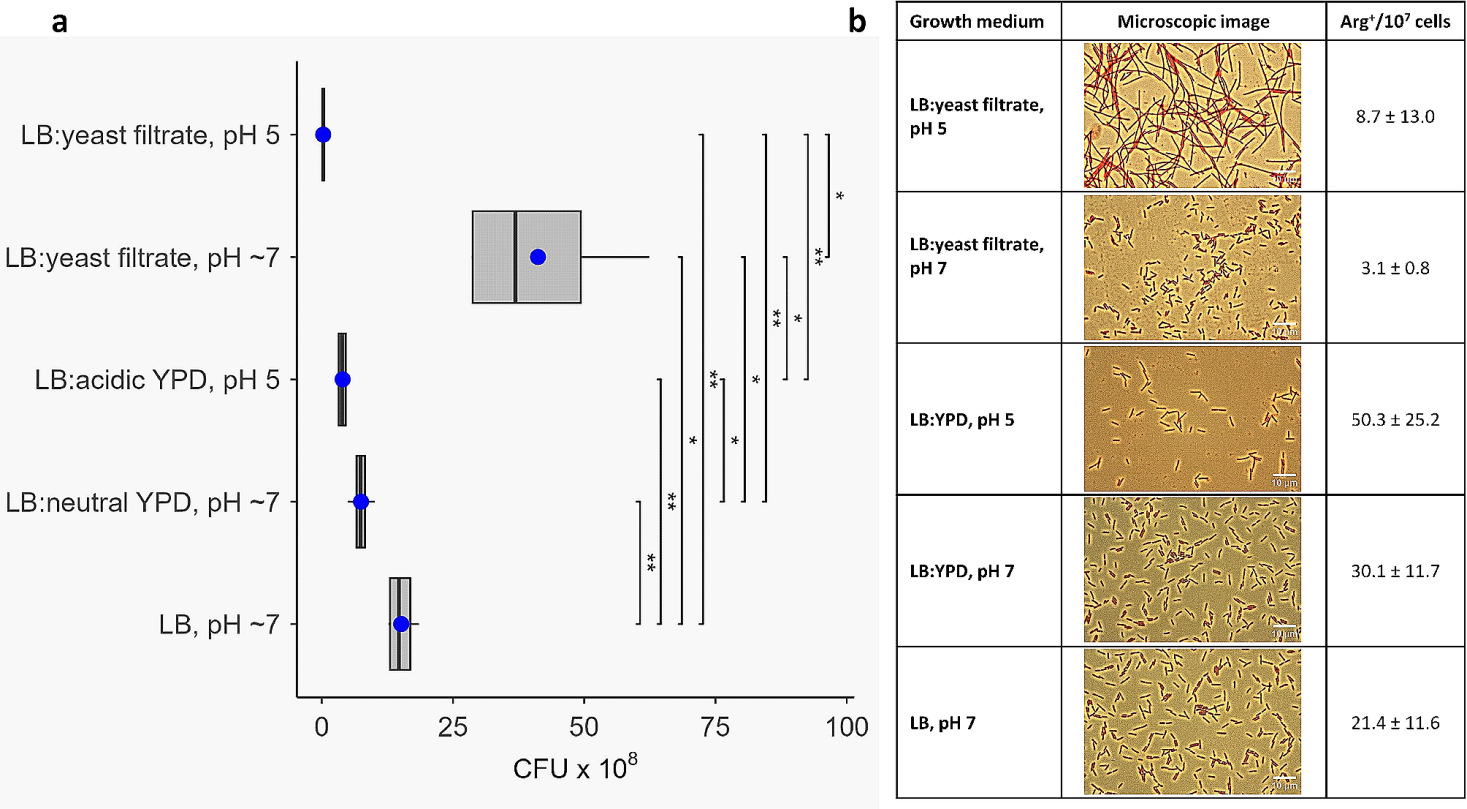
Effects of crude filtrates of *K. humilis* MAW1 cultures on the growth of *E. coli* strain AB1157. (**a**) Number of viable cells of *E. coli* strain AB1157 (CFU, colony forming units) after overnight growth under standard conditions (37 °C with shaking) in the following media: LB alone, or equal (1:1) mixtures of LB: neutral YPD (pH 7.0), LB: acidic YPD (pH 5.0), LB: culture filtrate after *K. humilis* MAW1 growth in acidic YPD (pH 4.5) or LB: culture filtrate after *K. humilis* MAW1 growth in neutral YPD (pH 7.0). Data represent the means ± SD from four biological replicates, the statistical significance of two-sided t-test is shown (0.05 ≥ *> 0.01 ≥ **> 0.001 ≥ ***); (**b**) Microscopic images of *E. coli* AB1157 stained with fuchsin (enhanced in Fiji) and the level of *argE3*→Arg^+^ reversion (mean ± SD from four biological replicates) after overnight growth under the aforementioned conditions

The overnight culture of *E. coli* AB1157 in LB medium contained ∼ 1.5 × 10^9^ cells/lm (Fig. 8a), which is typical for this strain. The number of bacteria decreased 2-fold to ∼ 0.75 × 10^9^ cells/ml or 3.75-fold to 0.4 × 10^9^ cells/ml when *E.coli* AB1157 was grown in 1:1 mixtures of LB: neutral (pH 7.0) YPD or LB: acidic (pH 5) YPD media, respectively (Fig. 8a). Since citrate-phosphate buffer was used as an acidifying agent, undissociated citric acid or pH 5 itself may cause some inhibition of bacterial growth. Surprisingly, after cultivation of *K. humilis* MAW1 in neutral (pH 7.0) YPD medium the yeast extract promoted *E. coli* AB1157 growth, since the number of bacteria in the overnight culture reached ∼ 4.1 × 10^9^ cells/ml, i.e. 2.7-fold and 5.5-fold higher in comparison to overnight cultures grown in LB alone and LB: neutral (pH 7.0) YPD, respectively. In comparison, after cultivation in acidic YPD the yeast extract caused strong inhibition of bacterial growth. The number of bacteria in the overnight culture in LB: yeast filtrate at pH ∼ 5.0 was ∼ 3.0 × 10^7^ cells/ml, corresponding to a 52-fold and 14-fold decrease compared to the overnight cultures in LB alone and LB: acidic YPD, respectively. Moreover, the number of bacteria grown in the mixtures of LB: yeast filtrate at acidic pH was decreased by 143-fold in comparison to that grown in LB: yeast filtrate at neutral pH (7.0).

Samples of the overnight *E. coli* AB1157 cultures were stained with basic fuchsin and inspected under a light microscope. In the presence of the crude filtrate of *K. humilis* MAW1 grown in acidic medium (pH 4.5), the bacteria formed filaments, whereas no significant filamentation was observed in any other incubation variant. A slight tendency to form filaments was also observed in *E. coli* AB1157 grown at acidic pH (Fig. 8b). It may be the result of undissociated citric acid from citrate-phosphate buffer and/or low pH. Filament formation is responsible for the lower colony numbers on solid medium, since one filament comprised of several cells produces only one colony.

The *E. coli* AB1157 strain bears the *argE3* (ochre) mutation which can revert to prototrophy (Arg^+^), so this strain has been used in studies on mutagenesis and induction of the SOS system, i.e. the bacterial response to DNA damage and the arrest of DNA synthesis [75, 76]. Induction of the SOS system is characterized by the filamentation of bacterial cells due to the inhibition of cell division and an increase in the level of mutations. Here, filament formation was not associated with mutation induction, as measured by the level of *argE3*→Arg^+^ reversions (Fig. 8b), which indicates that the yeast filtrates had no mutagenic effects.

### Genes encoding proteins with potential antimicrobial properties and accessory proteins in the *K. humilis* MAW1 genome

The assembled genome sequence of *K. humilis* MAW1 was surveyed for genes with potential antimicrobial activity using several characterized proteins as the query sequences in Blast searches [see Supplementary Table 1 in Additional file 1]. Using an E-value of < ∼ 1 and/or Query coverage of > ∼ 50% as the cut-off values, 9 potential orthologues were identified: P09807, P10410, A5A0Q7, B9WE14, G8B7 × 9, J7S410, J7S427, J8Q1Q0 and M3HTF7 [see Supplementary Table 1 in Additional file 1]. One of these proteins, J7S427, with query coverage (QC) and identical sites (IS) values of 98.76% and 63.90%, respectively, is a Vac14_Fig4_bd domain-containing protein. This participates in phosphatidylinositol biosynthesis as part of a class III phosphatidylinositol 3-kinase complex (PAS) [77–79]. The Vac14_Fig4_bd domain is responsible for the dynamic interconversion of PI3P and PI(3,5)P2p. Three other proteins with potential antimicrobial properties, G8B7 × 9, J7S410, and J8Q1Q0, contain a cellulase domain involved in hydrolyzing O-glycosyl compounds [80–83]. These genes were found in three contigs, each at approximately the same locations, suggesting quite a high degree of homology between these proteins, with QC values of 58.70–98.21% and IS values ranging from 22.70 to 65.10%. The arginase B9WE14, and M3HTF7, a U4/U5/U6 small nuclear ribonucleoprotein prp3, probably exert some anti-bacterial activity. Genes encoding 3 more directly toxic proteins were also identified in the *K. humilis* MAW1 genome. These were a killer toxin subunit γ, RF3, P09807 [84](QC 56.63%; IS 24.30%), HM-1 toxin, P10410, inhibiting β-1,3-glucan synthesis [85–87] (QC 59.20%; IS 7.00%) and an endo-β-1,3-glucanase, A5A0Q7 [88, 89], which is probably encoded by at least three genes (QC 69.87–92.95%; IS 26.60–80.00%) (Table 3).

**Table 3:** Results of tblastn searches of the *K. humilis* MAW1 genome assembly for genes/proteins with potential bacteriostatic activity, using known toxins as query sequences. Only significant results are shown (E-value < ∼ 1 and/or Query coverage > ∼ 50%). For full tblastn results, see Additional file 2

Query	Name	Bit-Score	E-value	Grade (%)	Query coverage (%)	Identical Sites (%)	Query start	Query end	Hit start	Hit end	Min Seq Length	Max Seq Length	Sequence Length
P09807	tig00000030	31.5722	2.63E-01	28.30	56.63	24.30	53	193	31,556	31,945	130	141	1,038,856
P10410	tig00006880	26.9498	1.79E + 00	29.60	59.20	27.00	3	76	1,590,164	1,589,961	68	74	2,307,406
A5A0Q7	tig00000003	60.8474	6.22E-10	42.10	84.29	27.10	36	298	1,732,878	1,732,156	241	263	2,170,539
	tig00000063	477.248	2.48E-135	86.50	92.95	80.00	23	312	15,756	16,625	290	290	373,102
	tig00000063	67.3958	6.82E-12	35.30	70.51	27.90	34	253	32,361	32,969	203	220	373,102
	tig00000071	55.0694	3.50E-08	34.90	69.87	26.60	34	251	286,146	286,769	208	218	489,712
B9WE14	tig00000028	66.6254	1.48E-11	32.80	65.56	25.80	120	395	574,768	573,929	276	280	956,737
G8B7 × 9	tig00000014	66.2402	2.08E-11	30.30	60.61	22.70	32	288	1,070,560	1,069,766	257	265	1,309,822
	tig00000122	427.172	5.40E-120	73.90	94.58	53.20	21	421	423,598	422,372	401	409	484,836
	tig00006880	294.278	4.29E-80	69.60	89.15	39.70	38	415	194,608	195,921	378	438	2,307,406
J7S410	tig00000014	64.6994	5.88E-11	29.40	58.70	25.20	42	294	1,070,539	1,069,769	253	257	1,309,822
	tig00000122	520.776	2.93E-148	77.20	91.65	62.70	37	431	423,559	422,375	395	395	484,836
	tig00006880	291.967	2.59E-79	70.00	90.02	38.70	40	427	194,605	195,924	388	440	2,307,406
J7S427	tig00000003	1071.23	0	81.30	98.76	63.90	1	874	634,814	637,375	854	874	2,170,539
J8Q1Q0	tig00000014	55.0694	4.86E-08	32.30	64.51	24.40	55	343	1,070,539	1,069,664	289	292	1,309,822
	tig00000122	610.527	2.90E-175	81.70	98.21	65.10	4	443	423,721	422,378	440	448	484,836
	tig00006880	259.996	1.11E-69	74.00	97.99	35.50	1	439	194,446	195,921	439	492	2,307,406
M3HTF7	tig00000003	72.4034	2.79E-13	19.40	38.85	27.40	261	429	334,996	335,520	169	175	2,170,539
	tig00000003	62.3882	3.35E-10	14.10	28.28	39.40	159	281	334,637	335,029	123	131	2,170,539

Other proteins that might confer resistance to or take part in the response to yeast toxins were also investigated [see Supplementary Table 2 in Additional file 1]. Potential KEX1 (P09231, P09620, Q4P8U8, Q6CKK4) and KEX2 (P13134) protease genes were identified (QC 50.34–77.65%; IS 28.40–67.30%) (Table 4). These proteases are involved in the posttranslational modification of precursors of the K1, K2 and K28 killer toxins and α-factor (mating pheromone) (see e.g [90–92]. Several genes were identified encoding KRE2 (P27809, A0A5P2U9Q3) and KRE5 (P22023, A0A5P2TZB3, W0TAU5), which are accessory proteins involved in the synthesis of β-1-6-D-glucan, a major structural polymer in the cell wall and target of killer toxins [93–96] (QC 41.94–99.55%; IS 29.60–63.70%). A gene encoding Fps1 was also detected (P23900) (QC 86.85%; IS 52.90%). This membrane glycerol uptake/efflux facilitator protein is a known interaction partner of killer toxins [97]. Another interesting protein, with at least two paralogues encoded in the *K. humilis* MAW1 genome, is KTI12 (P34253) (QC 56.87–100%; IS 58.10–65.00%), a target for *K. lactis* zymocin (see e.g [98, 99]. Lastly, there is a gene encoding Alg3 (P38179) (QC 49.34%; IS 57.50%), an α-1,3-mannosyltransferase sensitive to *Hansenula mrakii* HM-1 killer toxin [100, 101].

**Table 4:** Results of tblastn searches of the *K. humilis* MAW1 genome assembly for genes encoding potential proteins accompanying the toxic yeast phenotype, using protein sequences of known function or their close homologues as query sequences. Only significant results are shown (E-value < ∼ 1 and/or Query coverage > ∼ 50%). For full tblastn results, see Additional file 3

Query	Name	Bit-Score	E Value	Grade (%)	Query coverage (%)	Identical Sites (%)	Query start	Query end	Hit start	Hit end	Min Seq Length	Max Seq Length	Sequence Length
P09231	tig00000003	743.806	0	69.50	77.65	61.30	2	588	825,796	824,027	587	590	2,170,539
P09620	tig00000003	141.354	8.53E-38	50.20	50.34	29.70	67	433	1,712,380	1,713,402	341	367	2,170,539
	tig00006880	454.907	3.67E-128	57.60	65.29	45.60	28	503	1,901,441	1,902,916	476	492	2,307,406
P13134	tig00000003	848.195	0	69.80	72.36	67.30	17	605	825,766	824,015	584	589	2,170,539
	tig00000003	105.531	6.37E-23	37.40	25.06	48.60	583	786	824,084	823,476	203	204	2,170,539
P22023	tig00000121	447.588	0	48.20	46.37	40.70	733	1365	168,286	166,385	633	634	384,248
	tig00000121	302.753	0	37.00	24.03	47.60	414	741	169,305	168,274	328	344	384,248
	tig00000121	81.2629	0	29.50	9.08	34.60	292	415	169,652	169,299	118	124	384,248
	tig00000121	74.3294	1.89E-27	30.50	10.92	31.80	170	318	170,032	169,601	144	149	384,248
	tig00000121	67.781	1.89E-27	30.30	10.70	33.50	24	169	170,475	170,032	146	148	384,248
P23900	tig00000003	619.387	1.17E-177	69.90	86.85	52.90	77	657	1,612,573	1,614,420	581	616	2,170,539
P27809	tig00000003	373.244	7.73E-104	62.90	73.98	51.80	108	434	1,173,915	1,172,902	327	338	2,170,539
	tig00000003	155.606	8.73E-49	46.00	42.08	37.40	179	364	1,188,505	1,187,921	186	195	2,170,539
	tig00000003	56.225	8.73E-49	31.00	11.99	45.30	123	175	1,188,677	1,188,519	53	53	2,170,539
	tig00000003	51.6026	5.69E-07	6.30	12.67	44.60	352	407	1,187,960	1,187,811	50	56	2,170,539
	tig00000010	247.669	5.64E-66	59.70	69.46	38.90	101	407	12,951	13,895	307	315	30,868
	tig00000010	197.978	5.67E-51	57.20	64.48	39.20	126	410	27,855	28,694	280	285	30,868
	tig00000019	433.335	6.96E-122	66.10	72.40	59.80	120	439	141,545	140,583	320	321	1,373,199
	tig00000019	295.434	2.01E-80	59.60	69.23	46.00	116	421	1,083,558	1,084,523	306	322	1,373,199
	tig00000021	558.525	1.29E-159	79.40	99.55	59.20	1	440	351,448	350,105	440	448	1,118,589
	tig00000028	290.041	1.07E-78	59.20	68.33	43.70	113	414	763,024	762,077	302	316	956,737
	tig00000028	191.815	3.24E-49	63.00	76.02	29.60	104	439	472,509	473,687	336	393	956,737
	tig00000030	227.639	5.70E-60	56.20	62.44	37.40	120	395	472,073	471,054	276	340	1,038,856
	tig00006880	223.787	7.45E-59	56.80	63.57	35.70	115	395	727,074	728,132	281	353	2,307,406
P34253	tig00000005	369.392	7.88E-103	79.00	100.00	58.10	1	313	35,042	34,110	311	313	39,846
	tig00006880	245.743	5.85E-86	60.90	56.87	65.00	9	186	550,153	550,689	178	179	2,307,406
	tig00006880	65.4698	5.85E-86	36.30	20.13	52.40	197	259	550,719	550,901	61	63	2,307,406
	tig00006880	45.8246	5.85E-86	32.20	13.10	51.20	262	302	550,907	551,026	40	41	2,307,406
P38179	tig00000030	268.085	1.11E-123	53.40	49.34	57.50	3	228	643,978	643,310	223	226	1,038,856
	tig00000030	194.126	1.11E-123	46.10	42.14	49.00	222	414	643,331	642,738	193	198	1,038,856
Q4P8U8	tig00000003	148.673	5.85E-36	52.80	55.56	28.40	65	429	1,712,386	1,713,411	342	365	2,170,539
	tig00006880	257.299	1.10E-68	57.20	64.38	35.30	47	469	1,901,501	1,902,859	423	453	2,307,406
Q6CKK4	tig00000003	150.214	1.31E-41	53.30	56.70	30.60	67	430	1,712,389	1,713,390	334	364	2,170,539
	tig00000003	38.5058	1.31E-41	28.30	6.70	41.90	451	493	1,713,435	1,713,563	43	43	2,170,539
	tig00006880	405.601	2.36E-113	62.50	75.08	43.10	27	508	1,901,444	1,902,925	482	494	2,307,406
A0A5P2TZB3	tig00000121	244.202	3.73E-127	46.00	41.94	30.20	715	1237	168,229	166,544	523	562	384,248
	tig00000121	157.532	3.73E-127	36.90	23.90	29.50	388	685	169,305	168,349	298	319	384,248
	tig00000121	49.2914	3.73E-127	28.60	7.30	32.00	299	389	169,589	169,299	91	97	384,248
	tig00000121	47.7506	3.73E-127	30.10	10.18	27.50	162	288	170,032	169,625	127	136	384,248
	tig00000121	41.2022	3.73E-127	26.80	3.69	37.00	116	161	170,169	170,032	46	46	384,248
A0A5P2U9Q3	tig00000003	369.777	9.58E-103	62.90	73.54	52.20	104	417	1,173,852	1,172,899	314	318	2,170,539
	tig00000003	160.614	1.59E-51	46.40	42.86	38.50	164	346	1,188,499	1,187,921	183	193	2,170,539
	tig00000003	60.4622	1.59E-51	32.10	14.29	47.50	107	167	1,188,677	1,188,495	61	61	2,170,539
	tig00000003	49.6766	2.10E-06	6.40	12.88	43.60	334	388	1,187,960	1,187,802	53	55	2,170,539
	tig00000010	253.447	9.28E-68	59.30	68.62	41.10	96	388	12,990	13,904	293	305	30,868
	tig00000010	180.259	9.90E-46	58.30	66.51	35.10	108	391	27,849	28,691	281	284	30,868
	tig00000019	409.453	1.17E-114	65.90	75.64	56.10	101	423	141,554	140,577	323	326	1,373,199
	tig00000019	285.419	2.01E-77	63.50	77.05	42.10	98	426	1,083,552	1,084,589	329	346	1,373,199
	tig00000021	457.988	2.42E-129	69.30	74.94	63.70	104	423	351,067	350,102	320	322	1,118,589
	tig00000028	275.018	3.26E-74	67.30	84.54	38.30	31	391	763,222	762,092	361	377	956,737
	tig00000028	176.022	1.88E-44	67.90	85.71	27.20	54	419	472,371	473,681	366	437	956,737
	tig00000030	204.527	5.42E-53	57.10	64.17	34.40	104	377	472,073	471,054	274	340	1,038,856
	tig00006880	210.69	7.25E-55	56.70	63.47	36.20	107	377	727,098	728,132	271	345	2,307,406
W0TAU5	tig00000121	224.942	3.05E-120	46.80	43.60	30.10	696	1240	168,274	166,532	545	581	384,248
	tig00000121	152.14	3.05E-120	36.80	23.52	31.70	389	682	169,305	168,355	294	317	384,248
	tig00000121	51.2174	3.05E-120	29.90	9.84	29.10	163	285	170,032	169,637	123	132	384,248
	tig00000121	49.2914	3.05E-120	28.80	7.68	31.90	295	390	169,637	169,299	96	113	384,248
	tig00000121	38.891	3.05E-120	26.80	3.52	29.50	119	162	170,163	170,032	44	44	384,248

## Discussion

### Origin and characteristics of the selected phenotypic properties of *K. humilis* MAW1

The newly identified and characterized *K. humilis* strain MAW1 was isolated from a dark fermentation bioreactor processing sugar beet molasses. In this environment it constituted a kind of “infectious” agent for the hydrogen-yielding bacterial community. This yeast presumably originated from sugar beet. Microscopic examination of spent sugar beet transport water, raw juice from the initial stages of sugar beet processing, sugar beet leaves, and sugar beet pulp have revealed large quantities of yeast in these materials (unpublished observations).

*K. humilis* MAW1 is most similar to other *K. humilis* strains, especially YMX004033, KAHU0_CLIB1323v1, all isolated from naturally fermented foods. The current demand for healthy functional foods containing microorganisms that are beneficial for the human microbiome has resulted in a significant increase in research focused on natural sourdoughs, naturally fermented artisanal food products, and silage as a feed for livestock. A number of studies strongly indicate that *K. humilis* is one of the dominant microorganisms in various natural (type I) sourdoughs (with no added starter culture) and naturally fermented foods, along with lactic acid bacteria [17, 102–104]. The presence of *K. humilis* in natural sourdoughs and the likely primary source of *K. humilis* MAW1 confirm that these strains are associated with plants, cereals, vegetables and fruits. This is also consistent with the fact that most species of the *Kazachstania* genus have been isolated from soils.

To elucidate the metabolic characteristics of *K. humilis* MAW1 for comparison with other *K. humilis* strains, we used the Phenotype MicroArray system (Biolog Inc., USA). We believe that this is the first report of the system being used to characterize *K. humilis*. Presented here analysis showed that *K. humilis* MAW1 is capable of metabolizing a number of short-chain fatty acids, the most common acid fermentation products, and polyalcohols, as well as amino acids. The ability to utilize specific monosaccharides and disaccharides as carbon sources are consistent with previous data for this species. *K. humilis* MAW1 is able to metabolize glucose, galactose, sucrose and trehalose, some β-glucosides and glycogen. However, it cannot use lactose, raffinose, maltose, and the most of the pentoses analysed. This metabolic profile indicates that molasses-fed dark fermentation bioreactors are a highly attractive environment for this species. With regard to the utilization of sucrose and raffinose by *K. humilis*, the data provided by different authors are contradictory. Two studies determined that *K. humilis* was unable to ferment sucrose. The first one is the study of Nel and Walt (1968) who first isolated the species from Bantu beer and described it as *Torulopsis humilis* [12], the second study relates to the *K. humilis* YMX004033 strain isolated from agave fermentation [17, 18]. Yarrow (1978) [105] shows ability of *Candida milleri* (now *K. humilis*) to ferment both sucrose and raffinose.

An examination of the metabolic activity of *K. humilis* MAW1 under various stress conditions might assist the search for agents that could be added to bioreactors to inhibit yeast growth without influencing the dark fermentation process. A possible candidate might be sodium lactate, especially since its conversion to butyrate is a relevant pathway for hydrogen production [106].

In the phenotypic analysis of *K. humilis* MAW1, an extremely interesting topic is its ability to inhibit bacterial growth. Therefore, one of the goals of genome sequencing was the identification of factors likely to participate in the antibacterial activities.

### Phylogenetic characterization of *K. humilis *MAW1

We performed genome sequencing of diploid state new species of *K. humilis* MAW1, giving an advantage of heterogeneity studies. The general rule for new species classification of more than 1% difference in D1-D2 region and 1–2% in ITS region allowed to include the new species to *K. humilis* comprising distinct clade in the *Kazachstania* genus [107, 108]. The eight longest contigs measured more than 0.9 Mbp and many of them presented potential telomeric repeats making them good candidates for chromosomes. Indeed, there were 200,425 potential SNPs (14.9 per kb) and 9516 INDELs (0.7 per kb), which is quite comparable with other *Kazachstania* species, e.g. *K. servazzii* UCD13 showed the presence of 73,500 SNPs and 9400 IDNELs and *K. servazzii* UCD335, 3750 SNPs and 960 INDELs [109].

The genetic organization of MAT locus in *K. humilis* MAW1 and the other selected *Kazachstania* species makes it more similar to *K. humilis* YMX004033 and *K. humilis* KAHU0 in that they show the presence of one MATA1 or MATALHPA1/MATALPHA2 gene(s) only, in the genetic context of *S. cerevsiae* S288C, but HMl and HMR loci. The lack of potential HO endonuclease raises the question of the possibility of mating type switching. A similar phenomenon was shown for *K. africana* CBS 2517 having both MAT loci but no HO endonuclease [110]. Taking this into account, the *K. humilis* MAW1 was not able to produce spores for three weeks in the sporulation medium. The sporulation ability in the *Kazachstania* genus is very divergent. They can produce 1 to 16 ascospores [111]. On the other hand, *K. slooffiae* was not able to produce ascospores under different conditions [112] with one isolate sporulating, similarly for *K. menglunensis* [113]. Generally, *K. humilis* and *K. pseudohumilis* are considered as an asexual state [11].

The *Saccharomycetaceae* represent a very divergent yeast family. The killer toxin KHR1 was found in particular *S. cerevisiae* species but not in *S. cerevisiae* S288C [114]. This and similar phenomena make similar studies very valuable, widening the knowledge of genetics and physiology of this relevant yeast family.

### Inhibition of bacterial growth by *K. humilis* MAW1 in the light of its genome and the killer activities of other yeasts

One important trait, at least for some yeast species, is their ability to retard the growth of other microorganisms. However, so far there has been no direct evidence of antimicrobial activity or the production of killer toxins by *K. humilis.* This yeast has never been discussed in the context of antibacterial properties or as a producer of killer toxins [19, 22, 23]. Several studies have focused on the association between *K. humilis* and *Fructilactobacillus sanfranciscensis* as relevant components of sourdoughs [115–117]. Recent high-throughput DNA sequencing and metabolomics analyses of natural sourdoughs have been aimed at identifying the key players, understanding the dynamics of microbial communities during the fermentation process, and defining the correlation between microorganisms and metabolites, and the final product quality [116, 117]. These studies emphasized the stability of sourdough consortia, which probably results from mutualistic interaction between the microorganisms. The stimulation of bacterial growth caused by the crude filtrate after *K. humilis* MAW1 was cultured in neutral (pH 7.0) conditions, demonstrated here and in our previous study [19], could be an example of mutualistic interaction between yeasts and bacteria where yeasts are a source of nutritive compounds such as vitamins, amino acids, organic acids supporting bacterial growth. However, another as-yet unrecognized factor, such as the inhibitory effect on bacterial growth by yeasts, mainly *K. humilis*, may also be responsible for the maintenance of sourdoughs’ stability.

Our previous study provided clear evidence of the killer activity of the MAW1 isolate (now *K. humilis* MAW1) in acidic environments against a consortium of dark fermentation bacteria as well as pure cultures of various tester bacterial strains, both Gram-negative (*Klebsiella oxytoca, Citrobacter freundii, Escherichia coli* and *Pseudomonas putida*) and Gram-positive (*Bacillus megaterium, Clostridium butyricum*, strains of *Leuconostoc mesenteroides*, *Lactobacillus casei*, and *L. plantarum* isolated from milk and dairy products) [19]. This study investigates the effects of *K. humilis* MAW1 on *E. coli* K12 strain AB1157, a well characterized model bacterial strain. This strain has been used to search for links between survival, mutation induction and filaments formation after treatment with different agents [37]. Performed here analysis, including bacterial cell counts to monitor growth and microscopic examination of cell morphology, revealed that the antimicrobial activity of *K. humilis* MAW1 involves disturbances in bacterial cell division manifested by filamentation. It was not a matter of low pH since the relevant tests were performed. Nevertheless, a slight inhibitory effect of low pH and/or undissociated citric acid from citrate-phosphate buffer should be also considered. Filament formation is one of the characteristic features of bacterial growth inhibition after exposure to stress factors [118, 119]. However, in the case of crude yeast filtrate, it is not associated with mutation induction, at least as measured using the *argE3*→Arg^+^ reversion system. The similar interactions were also shown for *K. slooffiae* producing more biofilm in the presence of growth medium from *Lactobacillus acdiphilus*, with the opposite when the supernatant of growth medium from *Enterococcus faecalis* was used [112].

Bacterial cell division is determined by a multiprotein complex called the divisome. The main role is played by the protein FtsZ – a cytoplasmic tubulin-like GTP hydrolase that associates with the cytoplasmic membrane via the FtsA and ZipA proteins and forms a cytokinetic ring (Z ring). Additional key proteins are FtsK, FtsQ, FtsL, FtsB, FtsW, FtsI and FtsN [120]. Since FtsZ does not exist in higher eukaryotes, inhibitors of this protein have been discussed as potential antibacterial compounds [121]. In this regard, a search for interactions between metabolites secreted to the medium by *K. humilis* MAW1 and the divisome might be profitable.

The data presented here confirm our previous observations that a crude filtrate from *K. humilis* MAW1 cultured under acidic conditions can inhibit bacterial growth. This is also in accordance with the results of other studies, where tests on the killer activity of yeasts were performed in acidic environments, which were mainly explained by the pH-dependent binding of killer toxins to their cell wall receptors [27, 28, 122]. The previously proposed inhibitory agent β-1,3-glucosidase [19], considering its function in other species, is stable between pH 3.0-5.5, and loses its activity at higher pH. It is also noteworthy that fungicidal glycolipids are mainly active at quite low pH (∼ 4.5) [123–125]. Similarly, the killer toxin from *S. cerevisiae*, which inhibits the growth of some bacterial genera, including those capable of malolactic fermentation, is only active in acidic conditions [126].

A major reason for sequencing the *K. humilis* MAW1 genome was to search for genes encoding killer toxins. Using proteins with possible bacteriostatic activity to search the genome sequence, we identified several potential genes coding for polypeptides responsible for the hydrolysis of O-glycosyl compounds found in bacterial cell walls and for the kinase activity [80–83]. Exo-β-1,3-glucosidase activity is one of the best documented mechanisms of antimicrobial action, which involves hydrolysis of β-1,3-glucan, a component of bacterial cell walls [19]. Moreover, the *K. humilis* MAW1 genome encodes at least three potential endo-β-1,3-glucanases [88, 89]. Disorders in the structure of bacterial cell walls could also disturb the process of cell division which can lead to filamentation. Interestingly, *K. humilis* MAW1 was unable to metabolize X-β-D-glucoside when it was supplied with the substrate 5-Bromo-4-chloroindol-3-yl-β-D-glucopyranoside at neutral (pH 7.0) pH (PM10 MicroPlate). There are several possible explanations for this unexpected finding: (i) perhaps this activity develops exclusively in an acidic environment, (ii) the strain might not secrete the appropriate enzyme and lacks the ability to transport these substrates into the cell, or (iii) the strain may possess P-β-glucosidase activity so can only hydrolyze phosphorylated β-glucosides.

Other potential *K. humilis* MAW1 genes involved in sugar metabolism are those encoding a HM-1 homologue, an inhibitor of β-1,3-glucan synthesis, and toxin RF3 [84–87].

While we have focused on factors that potentially participate in the inhibitory activity of *K. humilis* MAW1, yeast killer toxins can have a broad specificity, so the inhibitory activity of this species may extend to other phyla. For example, KP4 was shown to inhibit mammalian L-type calcium channels [127], and the yeast *Metchnikowia pulcherrima* can retard the growth of many yeasts as well as bacteria [128].

Another group of proteins involved in the toxic phenotype of yeasts comprises those conferring resistance to autosecreted toxins. Some of them are proteases (e.g. KEX1 and KEX2) that cleave internalized toxins such as K1, K2 or K28 (see e.g [90–92]. Interestingly, the *K. humilis* MAW1 genome codes for three potential homologues of the KEX proteins. Several yeast killer toxins are encoded by genes located on extrachromosomal double-stranded (ds)RNA or dsDNA located in virus-like particles present in the cytoplasm. One example is toxin K28 which interrupts cell division by blocking DNA synthesis, and this can lead to filament formation by bacteria [20, 22, 23]. However, the procedure for DNA purification used in this study excluded the possibility of isolating extrachromosomal DNA and identifying extrachromosomal DNA-specific genes.

Yeast killer toxins, when secreted to the environment or when internalized by a heterologous cell, interact with specific molecules, often other proteins, residing in the cell wall matrix. Potential toxin targets encoded by multiple genes present in the genome of *K. humilis* MAW1 are KRE2/KRE5, responsible for the synthesis of β-1-6-D-glucan [93–96], and 1,3-mannosyltransferase Alg3 [100, 101]. Killer toxins that pass through the cell wall barrier interact with membrane proteins. One of them is Fps1, a membrane glycerol uptake/efflux facilitator protein [97], which is also encoded in the investigated genome. When they have gained entry to the cytoplasm, killer toxins interact with various proteins responsible for crucial metabolic processes, e.g. KTI12 involved in cell cycle arrest [98, 99]. The presence of the aforementioned genes renders *K. humilis* MAW1 a potential target for other yeasts with killer activity.

The advent of new technologies such as next generation sequencing as well as methods to culture novel microbial species have demonstrated the widespread nature of killer activity among yeasts [129–131].

Verification of relevance of the mentioned above proteins with potential antimicrobial properties would require examination of gene expression in *K. humilis* MAW1 by reverse transcription polymerase chain reaction (RT-PCR), detection of the proteins in the filtrates after yeast growth by Western Blot, and finally isolation of the proteins and tests with bacteria. Further studies are warranted.

### *K. humilis* MAW1 as a kind of cell factory in fermentation processes

**Fig. 9:**
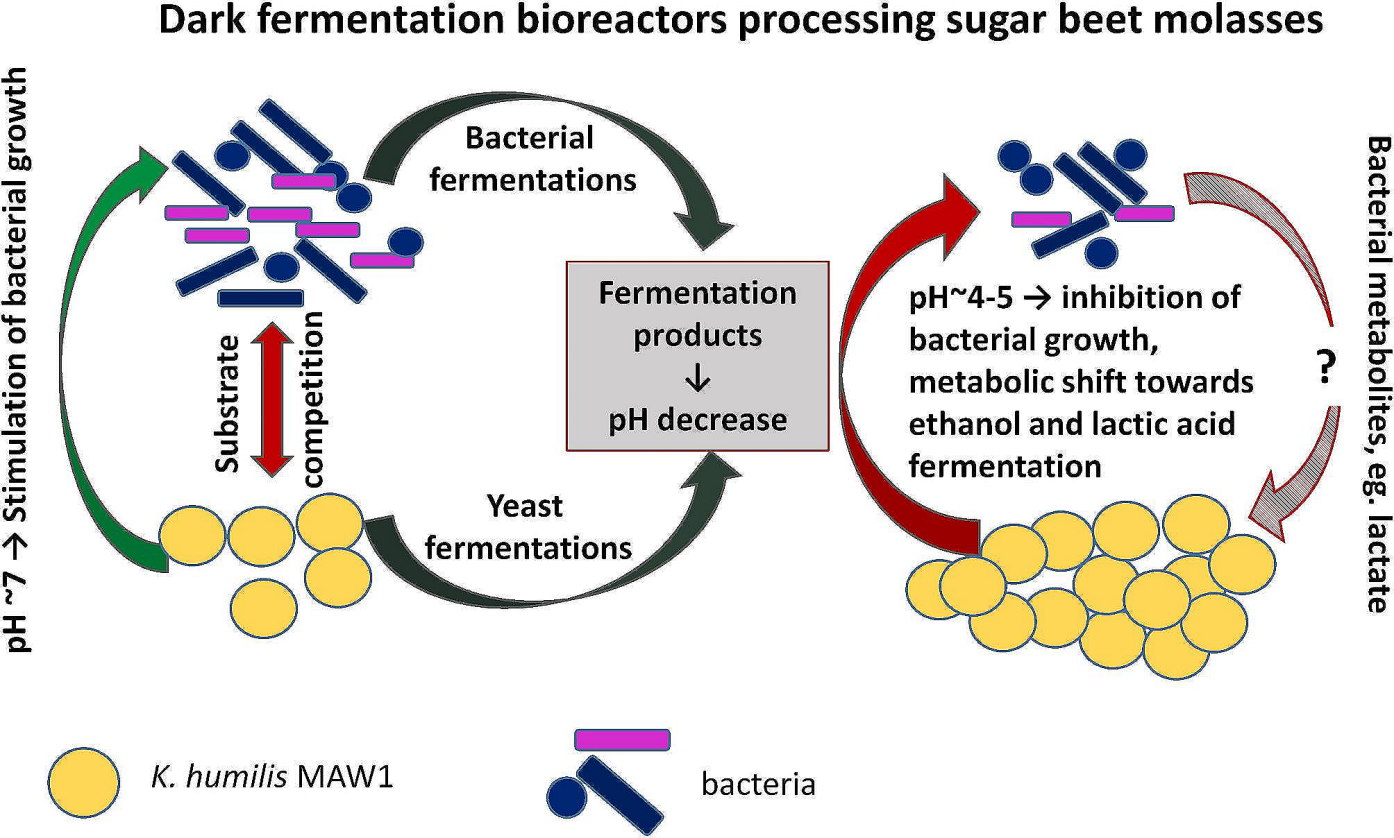
A possible scenario of *K. humilis* MAW1 acting as a specific cell factory and interacting with bacteria in the bioreactor of dark fermentation from which it has been previously isolated [19]

Summing up all the examined features of *K. humilis* MAW1 shown in this and previous [19] studies are presented in Fig. 9. The figure illustrates a possible scenario of *K. humilis* MAW1 acting as a specific cell factory in the bioreactor from which it was isolated. It was a dark fermentation bioreactor processing sugar beet molasses [19]. Under neutral conditions in the early stages of fermentation, yeast may provide nutrients for bacteria and stimulate bacterial growth. Both types of microorganisms compete for substrate, namely sucrose. Acidification of the environment by the products of fermentation induces mechanisms in yeasts that inhibit bacterial growth resulting in disruption of the bacterial community. Previously, we documented growth inhibition of hydrogen-producing *Citrobacter freundii, Klebsiella oxytoca*, and *Clostridium butyricum.* The scenario also includes metabolic shift in dark fermentation bioreactors towards ethanol and lactic acid fermentations observed previously [19]. These considerations omit the issue of bacterial effects on yeast. The sensitivity of *K. humilis* MAW1 to high concentrations of sodium lactate and the differentiated inhibition of LAB growth by *K. humilis* MAW1 shown previously [19] may indicate the importance of lactate in these interactions. All the issues presented in Fig. 9 can be subject to separate studies to recognize all the mechanisms in detail. The scenario can be extrapolated to other natural habitats of *K. humilis*, especially sourdoughs.

## Conclusions

The genome of *K. humilis* MAW1 has been sequenced and found to have a size of approximately 15.4 Mbp with 48.9% GC and 4856 genes. This species is most closely related to *K. humilis* – a yeast previously regarded as sourdough-specific as shown by phylogenetic analysis based on the 18 S-ITS-D1-D2 cluster. The species was sequenced in diploid state and its MAT locus organization is distinct from other *Kazachstania* species. *K. humilis* MAW1 was isolated from a dark fermentation bioreactor fed with molasses, and its source of origin was presumably sugar beet. Phenotype MicroArray analysis confirmed that molasses-fed dark fermentation bioreactors are a highly attractive environment for *K. humilis* MAW1 in terms of the carbon sources utilized and optimal pH. In an acidic environment *K. humilis* MAW1 displays inhibitory activity that involves disorder of cell division manifested by filament formation. Filamentation was not associated with the induction of mutations in *E. coli* K12 AB1157 measured by *argE3*→Arg^+^ reversion. Notably, under neutral (pH 7.0) conditions *K. humilis* MAW1 stimulates bacterial growth. Analysis of the *K. humilis* MAW1 genome identified sequences encoding proteins with potential inhibitory activity, including 1,3-β-glucan glycosidase and β-1,3-glucan synthesis inhibitor. Results of presented study contribute to better understanding of the physiology of the *K. humilis* species as a kind of cell factory in the machinery of fermentation processes and its functioning in microbial communities.

### Electronic supplementary material

Below is the link to the electronic supplementary material.

Supplementary Material 1

Supplementary Material 2

Supplementary Material 3

Supplementary Material 4

## Data Availability

The datasets generated during the current study are available in the NCBI Databases. The Illumina and Nanopore sequencing results were uploaded under the NCBI BioProject PRJNA785806 and two BioSamples: SAMN23578054 (for Illumina reads) and SAMN23578055 (for Nanopore reads).

## Abbreviations

CFU: colony forming units
LAB: lactic acid bacteria
LB: medium composed of 1% Bacto–tryptone, 0.5% yeast extract, 0.5% NaCl
YPD: medium composed of 1% Bacto yeast extract, 2% Bacto peptone, 2% dextrose
PM plates: Phenotype MicroArray plates
NCBI: National Center for Biotechnology Information
gDNA: genomic DNA
PCI: phenol–chloroform–isoamyl alcohol
INDELs: insertion and deletions
SNPs: single nucleotide polymorphisms
MAT locus: the mating–type locus
UPGMA: unweighted pair group method with arithmetic mean

## References

[1] Boekhout T, Aime MC, Begerow D, Gabaldón T, Heitman J, Kemler M, Khayhan K, Lachance M-A, Louis EJ, Sun S (2021). The evolving species concepts used for yeasts: from phenotypes and genomes to speciation networks. Fungal Divers.

[2] Boekhout T, Amend AS, El Baidouri F, Gabaldón T, Geml J, Mittelbach M, Robert V, Tan CS, Turchetti B, Vu D (2022). Trends in yeast diversity discovery. Fungal Divers.

[3] Wijayawardene NN, Hyde KD, Lumbsch HT, Liu JK, Maharachchikumbura SSN, Ekanayaka AH, Tian Q, Phookamsak R (2018). Outline of Ascomycota: 2017. Fungal Divers.

[4] Drumonde-Neves J, Fernandes T, Lima T, Pais C, Franco-Duarte R. Learning from 80 years of studies: a comprehensive catalogue of non-saccharomyces yeasts associated with viticulture and winemaking. FEMS Yeast Res 2021, 21(3). 10.1093/femsyr/foab017 33751099

[5] Jacques N, Sarilar V, Urien C, Lopes MR, Morais CG, Uetanabaro APT, Tinsley CR, Rosa CA, Sicard D, Casaregola S (2016). Three novel ascomycetous yeast species of the Kazachstania clade, Kazachstania saulgeensis sp. nov., Kazachstaniaserrabonitensis sp. nov. and Kazachstania australis sp. nov. Reassignment of Candida Humilis to Kazachstania Humilis f.a. comb. nov. and Candida Pseudohumilis to Kazachstania Pseudohumilis f.a. comb. Nov. Int J Syst Evol Microbiol.

[6] Kurtzman CP (2003). Phylogenetic circumscription of Saccharomyces, Kluyveromyces and other members of the Saccharomycetaceae, and the proposal of the new genera Lachancea, Nakaseomyces, Naumovia, Vanderwaltozyma and Zygotorulaspora. FEMS Yeast Res.

[7] Wolfe KH, Armisén D, Proux-Wera E, ÓhÉigeartaigh SS, Azam H, Gordon JL, Byrne KP. Clade- and species-specific features of genome evolution in the Saccharomycetaceae. FEMS Yeast Res 2015, 15(5). 10.1093/femsyr/fov035 PMC4629796 26066552

[8] Dimitrov R, Gouliamova D (2022). Phylogenetic cut-off values for species and genera discrimination of yeast. Acta Microbiol Bulg.

[9] Wu ZW, Bai FY (2005). Kazachstania aquatica sp. nov. and kazachstania solicola sp. nov., novel ascomycetous yeast species. Int J Syst Evol Microbiol.

[10] Chen R, Wei SC, Jiang YM, Wang QM, Bai FY (2010). Kazachstania taianensis sp. nov., a novel ascomycetous yeast species from orchard soil. Int J Syst Evol Microbiol.

[11] Kurtzman CP, Robnett CJ, Ward JM, Brayton C, Gorelick P, Walsh TJ (2005). Multigene phylogenetic analysis of pathogenic candida species in the Kazachstania (Arxiozyma) telluris complex and description of their ascosporic states as Kazachstania bovina sp. nov., K. heterogenica sp. nov., K. pintolopesii sp. nov., and K. Slooffiae sp. nov. J Clin Microbiol.

[12] Nel EE, van der Walt JP. Torulopsis Humilis, Sp. N. *Mycopathologia et mycologia applicata* 1968, 36(1):94–6. 10.1007/BF02057169 5748626

[13] Lhomme E, Urien C, Legrand J, Dousset X, Onno B, Sicard D (2016). Sourdough microbial community dynamics: an analysis during French organic bread-making processes. Food Microbiol.

[14] Safar SVB, Gomes FCO, Marques AR, Lachance MA, Rosa CA (2013). Kazachstania rupicola sp. nov., a yeast species isolated from water tanks of a bromeliad in Brazil. Int J Syst Evol Microbiol.

[15] Lu HZ, Cai Y, Wu ZW, Jia JH, Bai FY (2004). Kazachstania aerobia sp. nov., an ascomycetous yeast species from aerobically deteriorating corn silage. Int J Syst Evol Microbiol.

[16] Nisiotou AA, Nychas GJ (2008). Kazachstania Hellenica sp. nov., a novel ascomycetous yeast from a Botrytis-affected grape must fermentation. Int J Syst Evol Microbiol.

[17] Wittwer AE, Sicard D, Howell KS (2022). Kazachstania Humilis. Trends Microbiol.

[18] García-Ortega LF, Colón-González M, Sedeño I, Santiago-Garduño E, Avelar-Rivas JA, Kirchmayr MR, DeLuna A, Delaye L, Morales L, Mancera E (2022). Draft genome sequence of a Kazachstania humilis strain isolated from Agave Fermentation. Microbiol Resource Announcements.

[19] Detman A, Chojnacka A, Mielecki D, Błaszczyk MK, Sikora A (2018). Inhibition of hydrogen-yielding dark fermentation by ascomycetous yeasts. Int J Hydrogen Energ.

[20] Gil-Rodríguez AM, Garcia-Gutierrez E (2021). Antimicrobial mechanisms and applications of yeasts. Adv Appl Microbiol.

[21] Hatoum R, Labrie S, Fliss I (2012). Antimicrobial and probiotic properties of yeasts: from fundamental to novel applications. Front Microbiol.

[22] Klassen R, Schaffrath R, Buzzini P, Ganter P. Antagonistic interactions and killer yeasts. In; 2017: 1–46.

[23] Mannazzu I, Domizio P, Carboni G, Zara S, Zara G, Comitini F, Budroni M, Ciani M (2019). Yeast killer toxins: from ecological significance to application. Crit Rev Biotechnol.

[24] Al-Sahlany STG, Altemimi AB, Al-Manhel AJA, Niamah AK, Lakhssassi N, Ibrahim SA. Purification of bioactive peptide with Antimicrobial Properties produced by Saccharomyces cerevisiae. Foods (Basel Switzerland) 2020, 9(3). 10.3390/foods9030324 PMC7142856 32168785

[25] Cavalero DA, Cooper DG (2003). The effect of medium composition on the structure and physical state of sophorolipids produced by Candida Bombicola ATCC 22214. J Biotechnol.

[26] Dieuleveux V, Van Der Pyl D, Chataud J, Gueguen M (1998). Purification and characterization of anti-listeria compounds produced by Geotrichum candidum. Appl Environ Microbiol.

[27] Izgü F, Altinbay D (1997). Killer toxins of certain yeast strains have potential growth inhibitory activity on gram-positive pathogenic bacteria. Microbios.

[28] Meneghin M, Reis V, Antonini S (2010). Inhibition of Bacteria contaminating alcoholic fermentations by Killer yeasts. Brazilian Archives Biology Technol.

[29] Hipp SS, Lawton WD, Chen NC, Gaafar HA (1974). Inhibition of Neisseria gonorrhoeae by a factor produced by Candida albicans. Appl Microbiol.

[30] Goerges S, Koslowsky M, Velagic S, Borst N, Bockelmann W, Heller KJ, Scherer S (2011). Anti-listerial potential of food-borne yeasts in red smear cheese. Int Dairy J.

[31] Nagornaia SS, Zharova VP, Kotliar AN (1989). [Yeast antagonists in the normal microflora of the intestinal tract in the long-lived inhabitants of Abkhazia]. Mikrobiologicheskii Zhurnal.

[32] Vital S, Abranches J, Hagler A, Mendonça-Hagler L. Mycocinogenic yeasts isolated from Amazon soils of the Maracá Ecological Station, Roraima-Brazil. Brazilian J Microbiol 2002, 33.

[33] Martini AV, Rosini G (1989). Killer relationships within the yeast Genus kluyveromyces. Mycologia.

[34] Narvhus JA, Gadaga TH (2003). The role of interaction between yeasts and lactic acid bacteria in African fermented milks: a review. Int J Food Microbiol.

[35] Tachibana S, Chiou T-Y, Konishi M (2021). Machine learning modeling of the effects of media formulated with various yeast extracts on heterologous protein production in Escherichia coli. MicrobiologyOpen.

[36] Bachmann BJ. Escherichia coli and Salmonella typhimurium: cellular and molecular biology. In. Edited by Neidhardt FC. Washington, D.C.:: American Society for Microbiology; 1987.

[37] Sikora A, Grzesiuk E (2010). Reversion of argE3 to arg(+) in Escherichia coli AB1157 -an informative bacterial system for mutation detection. Acta Biochim Pol.

[38] Miller JH. Experiments in molecular genetics. Cold Spring Harbor Laboratory; 1972.

[39] Vogel HJ, Bonner DM (1956). Acetylornithinase of Escherichia coli: partial purification and some properties. J Biol Chem.

[40] Ausubel FM, Brent R, Kingston RE, Moore DE, Seidman J, Smith JA, Struhl K. Current protocols in Molecular Biology. John Wiley & Sons, Inc.; 2003.

[41] Hadley W. ggplot2: Elegant Graphics for Data Analysis. 2016.

[42] Ahlmann-Eltze C, Patil I. ggsignif: R Package for Displaying Significance Brackets for ‘ggplot2’; 2021.

[43] Schindelin J, Arganda-Carreras I, Frise E, Kaynig V, Longair M, Pietzsch T, Preibisch S, Rueden C, Saalfeld S, Schmid B (2012). Fiji: an open-source platform for biological-image analysis. Nat Methods.

[44] De Coster W, D’Hert S, Schultz DT, Cruts M, Van Broeckhoven C (2018). NanoPack: visualizing and processing long-read sequencing data. Bioinformatics.

[45] Bolger AM, Lohse M, Usadel B (2014). Trimmomatic: a flexible trimmer for Illumina sequence data. Bioinformatics.

[46] Loman NJ, Quinlan AR (2014). Poretools: a toolkit for analyzing nanopore sequence data. Bioinformatics.

[47] Marcais G, Kingsford C (2011). A fast, lock-free approach for efficient parallel counting of occurrences of k-mers. Bioinformatics.

[48] Vurture GW, Sedlazeck FJ, Nattestad M, Underwood CJ, Fang H, Gurtowski J, Schatz MC (2017). GenomeScope: fast reference-free genome profiling from short reads. Bioinformatics.

[49] Koren S, Walenz BP, Berlin K, Miller JR, Bergman NH, Phillippy AM (2017). Canu: scalable and accurate long-read assembly via adaptive k-mer weighting and repeat separation. Genome Res.

[50] Li H, Durbin R (2009). Fast and accurate short read alignment with Burrows-Wheeler transform. Bioinformatics.

[51] Li H, Handsaker B, Wysoker A, Fennell T, Ruan J, Homer N, Marth G, Abecasis G, Durbin R (2009). Genome Project Data Processing S: the sequence Alignment/Map format and SAMtools. Bioinformatics.

[52] Walker BJ, Abeel T, Shea T, Priest M, Abouelliel A, Sakthikumar S, Cuomo CA, Zeng Q, Wortman J, Young SK (2014). Pilon: an integrated tool for comprehensive microbial variant detection and genome assembly improvement. PLoS ONE.

[53] Bankevich A, Nurk S, Antipov D, Gurevich AA, Dvorkin M, Kulikov AS, Lesin VM, Nikolenko SI, Pham S, Prjibelski AD (2012). SPAdes: a new genome assembly algorithm and its applications to single-cell sequencing. J Comput Biol.

[54] Gurevich A, Saveliev V, Vyahhi N, Tesler G (2013). QUAST: quality assessment tool for genome assemblies. Bioinformatics.

[55] Chen S, Zhou Y, Chen Y, Gu J (2018). Fastp: an ultra-fast all-in-one FASTQ preprocessor. Bioinformatics.

[56] Li H. Aligning sequence reads, clone sequences and assembly contigs with BWA-MEM. *arXiv: Genomics* 2013.

[57] Brown M. González De la Rosa PM, Mark B: A Telomere Identification Toolkit. 2023.

[58] Team RC. R: a language and environment for statistical computing. R Foundation for Statistical Computing; 2014.

[59] Hoff KJ, Lange S, Lomsadze A, Borodovsky M, Stanke M (2016). BRAKER1: unsupervised RNA-Seq-based genome annotation with GeneMark-ET and AUGUSTUS. Bioinformatics.

[60] Ter-Hovhannisyan V, Lomsadze A, Chernoff YO, Borodovsky M (2008). Gene prediction in novel fungal genomes using an ab initio algorithm with unsupervised training. Genome Res.

[61] Lagesen K, Hallin P, Rodland EA, Staerfeldt HH, Rognes T, Ussery DW (2007). RNAmmer: consistent and rapid annotation of ribosomal RNA genes. Nucleic Acids Res.

[62] Lowe TM, Eddy SR (1997). tRNAscan-SE: a program for improved detection of transfer RNA genes in genomic sequence. Nucleic Acids Res.

[63] Todd RT, Braverman AL, Selmecki A (2018). Flow Cytometry Analysis of Fungal Ploidy. Curr Protoc Microbiol.

[64] Altschul SF, Madden TL, Schäffer AA, Zhang J, Zhang Z, Miller W, Lipman DJ (1997). Gapped BLAST and PSI-BLAST: a new generation of protein database search programs. Nucleic Acids Res.

[65] Kearse M, Moir R, Wilson A, Stones-Havas S, Cheung M, Sturrock S, Buxton S, Cooper A, Markowitz S, Duran C (2012). Geneious Basic: an integrated and extendable desktop software platform for the organization and analysis of sequence data. Bioinformatics.

[66] Danecek P, Bonfield JK, Liddle J, Marshall J, Ohan V, Pollard MO, Whitwham A, Keane T, McCarthy SA, Davies RM et al. Twelve years of SAMtools and BCFtools. Gigascience 2021, 10(2). 10.1093/gigascience/giab008 PMC7931819 33590861

[67] Tamura K, Nei M (1993). Estimation of the number of nucleotide substitutions in the control region of mitochondrial DNA in humans and chimpanzees. Mol Biol Evol.

[68] Sokal RR, Michener CD (1958). A statistical method for evaluating systematic relationships. Univ Kans Sci Bull.

[69] Johnson M, Zaretskaya I, Raytselis Y, Merezhuk Y, McGinnis S, Madden TL. NCBI BLAST: a better web interface. Nucleic Acids Res 2008, 36(Web Server issue):W5–9. 10.1093/nar/gkn201 PMC2447716 18440982

[70] White T, Bruns T, Lee S, Taylor J, Innis M, Gelfand D, Sninsky J (1990). Amplification and direct sequencing of fungal ribosomal RNA genes for phylogenetics. In.

[71] Larkin MA, Blackshields G, Brown NP, Chenna R, McGettigan PA, McWilliam H, Valentin F, Wallace IM, Wilm A, Lopez R (2007). Clustal W and Clustal X version 2.0. Bioinformatics.

[72] UniProt C (2021). UniProt: the universal protein knowledgebase in 2021. Nucleic Acids Res.

[73] Altschul SF, Gish W, Miller W, Myers EW, Lipman DJ (1990). Basic local alignment search tool. J Mol Biol.

[74] Coordinators NR (2016). Database resources of the National Center for Biotechnology Information. Nucleic Acids Res.

[75] Janion C, Sikora A, Nowosielska A, Grzesiuk E (2002). Induction of the SOS response in starved Escherichia coli. Environ Mol Mutagen.

[76] Janion C, Sikora A, Nowosielska A, Grzesiuk E (2003). E. Coli BW535, a triple mutant for the DNA repair genes xth, nth, and Nfo, chronically induces the SOS response. Environ Mol Mutagen.

[77] Jin N, Chow CY, Liu L, Zolov SN, Bronson R, Davisson M, Petersen JL, Zhang Y, Park S, Duex JE (2008). VAC14 nucleates a protein complex essential for the acute interconversion of PI3P and PI(3,5)P(2) in yeast and mouse. EMBO J.

[78] Michell RH, Dove SK (2009). A protein complex that regulates PtdIns(3,5)P2 levels. EMBO J.

[79] Sbrissa D, Ikonomov OC, Fenner H, Shisheva A (2008). ArPIKfyve homomeric and heteromeric interactions scaffold PIKfyve and Sac3 in a complex to promote PIKfyve activity and functionality. J Mol Biol.

[80] Butler G, Rasmussen MD, Lin MF, Santos MA, Sakthikumar S, Munro CA, Rheinbay E, Grabherr M, Forche A, Reedy JL (2009). Evolution of pathogenicity and sexual reproduction in eight Candida genomes. Nature.

[81] Gordon JL, Armisén D, Proux-Wéra E, ÓhÉigeartaigh SS, Byrne KP, Wolfe KH (2011). Evolutionary erosion of yeast sex chromosomes by mating-type switching accidents. Proc Natl Acad Sci USA.

[82] Guida A, Lindstädt C, Maguire SL, Ding C, Higgins DG, Corton NJ, Berriman M, Butler G (2011). Using RNA-seq to determine the transcriptional landscape and the hypoxic response of the pathogenic yeast Candida parapsilosis. BMC Genomics.

[83] Liti G, Nguyen Ba AN, Blythe M, Müller CA, Bergström A, Cubillos FA, Dafhnis-Calas F, Khoshraftar S, Malla S, Mehta N (2013). High quality de novo sequencing and assembly of the Saccharomyces arboricolus genome. BMC Genomics.

[84] Stark MJ, Mileham AJ, Romanos MA, Boyd A (1984). Nucleotide sequence and transcription analysis of a linear DNA plasmid associated with the killer character of the yeast Kluyveromyces Lactis. Nucleic Acids Res.

[85] Ashida S, Shimazaki T, Kitano K, Hara S (1983). New Killer Toxin of Hansenula Mrakii. Agric Biol Chem.

[86] Kasahara S, Ben Inoue S, Mio T, Yamada T, Nakajima T, Ichishima E, Furuichi Y, Yamada H (1994). Involvement of cell wall beta-glucan in the action of HM-1 killer toxin. FEBS Lett.

[87] Kimura T, Kitamoto N, Matsuoka K, Nakamura K, Iimura Y, Kito Y (1993). Isolation and nucleotide sequences of the genes encoding killer toxins from Hansenula mrakii and H. saturnus. Gene.

[88] Comitini F, Mannazzu I, Ciani M (2009). Tetrapisispora phaffii killer toxin is a highly specific β-glucanase that disrupts the integrity of the yeast cell wall. Microb Cell Fact.

[89] Comitini F, Pietro ND, Zacchi L, Mannazzu I, Ciani M (2004). Kluyveromyces phaffii killer toxin active against wine spoilage yeasts: purification and characterization. Microbiol (Reading).

[90] Latchinian-Sadek L, Thomas DY (1993). Expression, purification, and characterization of the yeast KEX1 gene product, a polypeptide precursor processing carboxypeptidase. J Biol Chem.

[91] Dmochowska A, Dignard D, Henning D, Thomas DY, Bussey H (1987). Yeast KEX1 gene encodes a putative protease with a carboxypeptidase B-like function involved in killer toxin and alpha-factor precursor processing. Cell.

[92] Wickner RB, Leibowitz MJ (1976). Two chromosomal genes required for killing expression in killer strains of Saccharomyces cerevisiae. Genetics.

[93] Meaden P, Hill K, Wagner J, Slipetz D, Sommer SS, Bussey H (1990). The yeast KRE5 gene encodes a probable endoplasmic reticulum protein required for (1- - - -6)-beta-D-glucan synthesis and normal cell growth. Mol Cell Biol.

[94] Hill K, Boone C, Goebl M, Puccia R, Sdicu AM, Bussey H (1992). Yeast KRE2 defines a new gene family encoding probable secretory proteins, and is required for the correct N-glycosylation of proteins. Genetics.

[95] Varela JA, Puricelli M, Ortiz-Merino RA, Giacomobono R, Braun-Galleani S, Wolfe KH, Morrissey JP (2019). Origin of Lactose Fermentation in Kluyveromyces lactis by Interspecies transfer of a neo-functionalized gene cluster during domestication. Curr Biol.

[96] Lertwattanasakul N, Kosaka T, Hosoyama A, Suzuki Y, Rodrussamee N, Matsutani M, Murata M, Fujimoto N, Suprayogi, Tsuchikane K (2015). Genetic basis of the highly efficient yeast Kluyveromyces marxianus: complete genome sequence and transcriptome analyses. Biotechnol Biofuels.

[97] Van Aelst L, Hohmann S, Zimmermann FK, Jans AW, Thevelein JM (1991). A yeast homologue of the bovine lens fibre MIP gene family complements the growth defect of a Saccharomyces cerevisiae mutant on fermentable sugars but not its defect in glucose-induced RAS-mediated cAMP signalling. Embo j.

[98] Butler AR, White JH, Folawiyo Y, Edlin A, Gardiner D, Stark MJ (1994). Two Saccharomyces cerevisiae genes which control sensitivity to G1 arrest induced by Kluyveromyces lactis toxin. Mol Cell Biol.

[99] Frohloff F, Fichtner L, Jablonowski D, Breunig KD, Schaffrath R (2001). Saccharomyces cerevisiae Elongator mutations confer resistance to the Kluyveromyces lactis zymocin. Embo j.

[100] Kimura T, Kitamoto N, Kito Y, Iimura Y, Shirai T, Komiyama T, Furuichi Y, Sakka K, Ohmiya K (1997). A novel yeast gene, RHK1, is involved in the synthesis of the cell wall receptor for the HM-1 killer toxin that inhibits beta-1,3-glucan synthesis. Mol Gen Genet.

[101] Sharma CB, Knauer R, Lehle L (2001). Biosynthesis of lipid-linked oligosaccharides in yeast: the ALG3 gene encodes the Dol-P-Man:Man5GlcNAc2-PP-Dol mannosyltransferase. Biol Chem.

[102] Comasio A, Verce M, Van Kerrebroeck S, De Vuyst L (2020). Diverse Microbial composition of Sourdoughs from different origins. Front Microbiol.

[103] Papalexandratou Z, Kaasik K, Kauffmann LV, Skorstengaard A, Bouillon G, Espensen JL, Hansen LH, Jakobsen RR, Blennow A, Krych L (2019). Linking cocoa varietals and microbial diversity of Nicaraguan fine cocoa bean fermentations and their impact on final cocoa quality appreciation. Int J Food Microbiol.

[104] Shang Z, Ye Z, Li M, Ren H, Cai S, Hu X, Yi J (2022). Dynamics of microbial communities, flavor, and physicochemical properties of pickled chayote during an industrial-scale natural fermentation: correlation between microorganisms and metabolites. Food Chem.

[105] Yarrow D (1978). Candida Milleri sp. nov. Int J Syst Evol Micr.

[106] Detman A, Laubitz D, Chojnacka A, Wiktorowska-Sowa E, Piotrowski J, Salamon A, Kaźmierczak W, Błaszczyk MK, Barberan A, Chen Y (2020). Dynamics and Complexity of Dark Fermentation Microbial communities Producing Hydrogen from Sugar Beet molasses in continuously operating packed Bed reactors. Front Microbiol.

[107] Kurtzman CP, Robnett CJ (1998). Three new insect-associated species of the yeast genus Candida. Can J Microbiol.

[108] Scorzetti G, Fell JW, Fonseca A, Statzell-Tallman A (2002). Systematics of basidiomycetous yeasts: a comparison of large subunit D1/D2 and internal transcribed spacer rDNA regions. FEMS Yeast Res.

[109] Faherty L, Lewis C, McElheron M, Garvey N, Duggan R, Shovlin B, Byrne TOC, O’Brien KP, Wolfe CE. KH: Draft genome sequences of two isolates of the yeast Kazachstania Servazzii recovered from Soil in Ireland. Microbiol Resource Announcements 2019, 8(44). 10.1128/MRA.01257-19 PMC6953512 31672753

[110] Wolfe KH, Armisen D, Proux-Wera E, OhEigeartaigh SS, Azam H, Gordon JL, Byrne KP (2015). Clade- and species-specific features of genome evolution in the Saccharomycetaceae. FEMS Yeast Res.

[111] Vaughan-Martini A, Lachance M-A, Kurtzman C. Kazachstania Zubkova (1971). In.; 2011: 439–470.

[112] Summers KL, Foster Frey J, Arfken AM. Characterization of Kazachstania slooffiae, a proposed commensal in the porcine gut. J Fungi (Basel) 2021, 7(2). 10.3390/jof7020146 PMC7922399 33671322

[113] Ke T, Zhai YC, Yan ZL, Hui FL (2019). Kazachstania jinghongensis sp. nov. and Kazachstania menglunensis f.a., sp. nov., two yeast species isolated from rotting wood. Int J Syst Evol Microbiol.

[114] Goto K, Iwase T, Kichise K, Kitano K, Totuka A, Obata T, Hara S (1990). Isolation and properties of a chromosome-dependent KHR killer toxin in Saccharomyces cerevisiae. Agric Biol Chem.

[115] Altilia S, Foschino R, Grassi S, Antoniani D, Dal Bello F, Vigentini I (2021). Investigating the growth kinetics in sourdough microbial associations. Food Microbiol.

[116] Carbonetto B, Nidelet T, Guezenec S, Perez M, Segond D, Sicard D. Interactions between Kazachstania humilis yeast species and lactic acid Bacteria in Sourdough. Microorganisms 2020, 8(2). 10.3390/microorganisms8020240 PMC7074792 32053958

[117] Wang X, Zhu X, Bi Y, Zhao R, Nie Y, Yuan W (2020). Dynamics of microbial community and changes of metabolites during production of type Ι sourdough steamed bread made by retarded sponge-dough method. Food Chem.

[118] Jones TH, Vail KM, McMullen LM (2013). Filament formation by foodborne bacteria under sublethal stress. Int J Food Microbiol.

[119] Wehrens M, Ershov D, Rozendaal R, Walker N, Schultz D, Kishony R, Levin PA, Tans SJ (2018). Size laws and Division Ring dynamics in Filamentous Escherichia coli cells. Curr Biol.

[120] Levin PA, Janakiraman A (2021). Localization, Assembly, and activation of the Escherichia coli Cell Division Machinery. EcoSal Plus.

[121] Kong Q, Yang Y (2021). Recent advances in antibacterial agents. Bioorg Med Chem Lett.

[122] Lopes CA, Sangorrín MP (2010). Optimization of killer assays for yeast selection protocols. Rev Argent Microbiol.

[123] Golubev VI, Tomashevskaia MA. [Characterization of mycocin secreted by Rhodotorula colostri (Castelli) lodder]. Izv Akademii nauk Ser Biol 2009(3):373–8. 19548622

[124] Morita T, Ishibashi Y, Fukuoka T, Imura T, Sakai H, Abe M, Kitamoto D (2011). Production of Glycolipid biosurfactants, cellobiose lipids, by Cryptococcus Humicola JCM 1461 and their interfacial properties. Biosci Biotechnol Biochem.

[125] Kulakovskaya TV, Kulakovskaya EV, Golubev WI (2003). ATP leakage from yeast cells treated by extracellular glycolipids of Pseudozyma fusiformata. FEMS Yeast Res.

[126] Dick KJ, Molan PC, Eschenbruch R (2015). The isolation from Saccharomyces cerevisiae of two antibacterial cationic proteins that inhibit malolactic bacteria. Vitis: J Grapevine Res.

[127] Muccilli S, Restuccia C (2015). Bioprotective Role of yeasts. Microorganisms.

[128] Viljoen B. Yeast Ecological Interactions. Yeast’Yeast, Yeast’Bacteria, Yeast’Fungi Interactions and Yeasts as Biocontrol Agents. In; 1970: 83–110.

[129] Vadkertiová R, Sláviková E (1995). Killer activity of yeasts isolated from the water environment. Can J Microbiol.

[130] Vadkertiová R, Sláviková E (2007). Killer activity of yeasts isolated from natural environments against some medically important Candida species. Pol J Microbiol.

[131] Buzzini P, Martini A (2000). Biodiversity of killer activity in yeasts isolated from the Brazilian rain forest. Can J Microbiol.

