# Supplementary material for: Unlocking the genome of the non-sourdough Kazachstania humilis MAW1: insights into inhibitory factors and phenotypic properties
Source: Microb Cell Fact. 2024 Apr 15;23:111. doi: 10.1186/s12934-024-02380-7 (PMC11017505; doi:10.1186/s12934-024-02380-7)
Supplement: Supplementary file 1 — Supplementary Material 1 [file 12934_2024_2380_MOESM1_ESM.pdf]

**Supplementary Table 1.** Selected yeast proteins with toxic activity against microorganisms, used for Blast searches of the *Kazachstania humilis* MAW1 genome assembly, grouped by general function, with the location of the coding sequence if not chromosomal DNA. The functions are mainly retrieved from UniProtKB [1].

| UniProtKB Accession | Name | Strain | Function | Reference |
|---|---|---|---|---|
| J7S427 | Vac14_Fig4_bd domain-containing protein | *Kazachstania naganishii* CBS 8797 | Phosphatidylinositol biosynthetic process; PAS complex | [2] |
| J7S410 | cellulase domain-containing protein | *Kazachstania naganishii* CBS 8797 | Hydrolase activity, hydrolyzing O-glycosyl compounds | [2] |
| J8Q1Q0 | Exg1p | *Saccharomyces arboricola* H-6 | Hydrolase activity, hydrolyzing O-glycosyl compounds;<br>cellular glucan metabolic process;<br>extracellular region;<br>fungal-type cell wall | [3] |
| G8B7X9 | cellulase domain-containing protein, XOG1 | *Candida parapsilosis* CDC317 | Hydrolase activity, hydrolyzing O-glycosyl compounds;<br>carbohydrate metabolic process;<br>cellular anatomical entity | |
| B9WE14 | Arginase | *Candida dubliniensis* CD36 | Arginase activity;<br>metal ion binding;<br>regulation of nitrogen compound metabolic process;<br>regulation of primary metabolic process | |
| P09807 | Killer toxin subunit γ, RF3 | *Kluyveromyces lactis* CBS2359/152;<br>pGKL1 / k1 plasmid | Toxin | [4] |
| M3HTF7 | U4/U5/U6 small nuclear ribonucleoprotein prp3 | *Candida maltose* Xu316 | mRNA splicing, via spliceosome;<br>U4/U6 x U5 tri-snRNP complex | |
| P01546 | K1, M1-1 protoxin | *S. cerevisiae*;<br>(ScV-M1) TF325 dsDNA | Ionophoric toxin; recognizes β-1,6-D-glucan | [5-9] |
| E9PA29 | K2, K2 killer toxin | *S. cerevisiae*;<br>M2-1 dsDNA | Ionophoric toxin; recognizes β-1,6-D-glucan | [5, 10, 11] |
| P22313 | killer toxin KHR, KHR1 | *Saccharomyces cerevisiae* | Toxin | [12] |
| P39690 | killer toxin KHS, KHS1 | *Saccharomyces cerevisiae* | Toxin | [13] |
| Q00569 | K5, panomycocin | *Wickerhamomyces anomalus* NCYC434, BCA15, BCU24, BS91, DBVPG 3003, ATCC 96603 | Hydrolysis of β-1,6-/β-1,3-glucan | [14-18] |
| A5A0Q7 | KpKt, endo-b-1,3-glucanase | *Tetrapisispora Phaffii* DBVPG6076 | Hydrolysis β-1,6-/β-1,3-glucan | [19, 20] |
| Q7LZU3 | K28 / Killer toxin K28 | *S. cerevisiae*;<br>M28 virus dsDNA | Inhibits DNA synthesis; recognizes α-1,3-mannoprotein | [6, 21] |

| | | | | |
|---|---|---|---|---|
| P09805 | zymocin, killer toxin subunits alpha/beta | *Kluyveromyces lactis* NRRL Y-1140; pGKl-1 dsDNA | Exochitinase activity; $tRNA^{Glu}$ cleavage; cell-cycle perturbation | [4, 22-24] |
| Q707V3 | PaT / Fam-a protein | *Millerozyma acaciae* NRRLY18665; pPac1-2 linear plasmids | Exochitinase activity; $tRNA^{Gln}$ cleavage | [25, 26] |
| Q7Z8R9 (γ subunit) Q7Z8R6 (α and β subunits) | PiT, Egg protein | *Babjeviella inositovora* NRRL Y-18709*;* pPin1-3 killer plasmid | rRNA cleavage; binds to chitin | [27, 28] |
| Q90121 | KP4, KP4 killer toxin | *Ustilago maydis*; virus P4 dsRNA | Inhibits voltage-gated calcium channels | [29, 30] |
| P16948 | KP6 | *Ustilago maydis*; virus P6 | $K^+$ depletion | [31] |
| P19972 | SMKT, SMK1, salt-mediated killer protoxin 1 | *Millerozyma farinosa* KK1 | Membrane permeabilization | [32, 33] |
| Q8NJU1 | Zygocin, killer toxin zygocin | *Zygosaccharomyces bailii* 412 | Membrane permeabilization, Mannoprotein target | [34, 35] |
| P10410 | HMK, HM-1, Killer toxin HM-1 | *Cyberlindnera mrakii* IFO0895 | Inhibits β-1,3-glucan synthesis | [36-38] |
| D6NTN8 | Klus | *Saccharomyces cerevisiae* dsRNA | Unknown | [39] |

**Supplementary Table 2.** Selected proteins perfoming accompanying roles in the toxic activity of yeasts against other microorganisms, used for Blast searches of the *Kazachstania humilis* MAW1 genome assembly, with the location of the coding sequence if not chromosomal DNA. The functions are mainly retrieved from UniProtKB [1].

| UniProtKB Accession | Name | Strain | Function | Reference |
|---|---|---|---|---|
| P09806<br>P05469 | RF4 | *Kluyveromyces lactis* CBS2359/152 pGKL1 and pGKL2 plasmids | Immunity determinant | [4, 24, 40-42] |
| P17260 | KRE1, killer toxin-resistance protein 1 | *Saccharomyces cerevisiae* | Acts as the plasma membrane receptor for the yeast K1 viral toxin | [43-47] |
| P27809 | KRE2, glycolipid 2-alpha-mannosyltransferase | *Saccharomyces cerevisiae* | | [48-50] |
| P22023 | KRE5, killer toxin-resistance protein 5 | *Saccharomyces cerevisiae* | Required for β-1-6-D-glucan synthesis and normal cell growth | [51] |
| W0TH48 | KRE1 | *Kluyveromyces marxianus* DMKU3-1042 | | [52] |
| W0TAU5 | KRE5, killer toxin-resistance protein 5 | *Kluyveromyces marxianus* DMKU3-1042 | | [52] |
| A0A5P2U3X4 | KRE1 | *Kluyveromyces lactis* CBS 2105 | | [53] |
| A0A5P2U364 | KRE1 | *Kluyveromyces lactis* CBS 2105 | | [53] |
| A0A5P2U6V5 | KRE1 | *Kluyveromyces lactis* CBS 2105 | | [53] |
| A0A5P2U8G6 | KRE1 | *Kluyveromyces lactis* CBS 2105 | | [53] |
| A0A5P2U9Q3 | Kre2/Ktr6 | *Kluyveromyces lactis* CBS 2105 | | [53] |
| A0A5P2TZB3 | Kre5 | *Kluyveromyces lactis* CBS 2105 | | [53] |
| P43497 | Cwp2p | *Saccharomyces cerevisiae* | Plasma membrane receptor for *Pichia membranifaciens* killer toxin | [54, 55] |
| P34253 | KTI12 | *Saccharomyces cerevisiae* | Regulates elongator complex γ-toxin target (TOT) activity, resulting in G1 block by *K. lactis* toxin zymocin (pGKL1 killer toxin) | [56-67] |
| P09620 | KEX1, pheromone-processing carboxypeptidase KEX1 | *Saccharomyces cerevisiae* | Protease with a carboxypeptidase B-like function involved in the C-terminal processing of the lysine and arginine residues from the precursors of K1, K2 and K28 killer toxins and α-factor (mating pheromone) | [68-75] |
| P13134 | KEX2, Kexin | *Saccharomyces cerevisiae* | Processing of precursors of α-factors and killer toxin | [76-78] |
| P09231 | KEX1, Protease KEX1 | *Kluyveromyces lactis* NRRL Y-1140 | Probably involved in the processing of the precursor of m1-toxin and alpha-factor | [79] |

| | | | | |
|---|---|---|---|---|
| Q6CKK4 | KEX1, pheromone-processing carboxypeptidase KEX1 | *Kluyveromyces lactis* NRRL Y-1140 | Protease with a carboxypeptidase B-like function involved in the C-terminal processing of the lysine and arginine residues from protein precursors | |
| Q4P8U8 | KEX1, Pheromone-processing carboxypeptidase KEX1 | *Ustilago maydis* 521 | Protease with a carboxypeptidase B-like function involved in the C-terminal processing of the lysine and arginine residues from protein precursors; promotes cell fusion and is involved in the programmed cell death (by similarity) | |
| P38179 | Alg3, Dol-P-Man:Man(5)GlcNAc(2)-PP-Dol α-1,3-mannosyltransferase | *Saccharomyces cerevisiae* | Adds the first Dol-P-Man derived mannose in an α-1,3 linkage to Man5GlcNAc2-PP-Dol; sensitive to *H. mrakii* HM-1 killer toxin | [80, 81] |
| P23900 | Fps1, glycerol uptake/efflux facilitator protein | *Saccharomyces cerevisiae* | Channel protein for glycerol; has a role in both glycerol influx and efflux; plays a role in osmoregulation: under osmotic stress the channel is apparently closed to allow accumulation of glycerol in the cell under hyperosmotic conditions | [82] |
| P18414 | Erd2 | *Saccharomyces cerevisiae* | Required for the retention of luminal endoplasmic reticulum proteins; determines the specificity of the luminal ER protein retention system; also required for normal vesicular traffic through the Golgi; this receptor strongly recognizes H-D-E-L and weakly recognizes D-D-E-L and K-D-E-L | [83-86] |

**Supplementary Table 3.** Statistics for Illumina PE reads used for the assembly of the *K. humilis* MAW1 genome, before and after filtration (F – forward, R – reverse).

| Statistics | F raw reads | F clean reads | R raw reads | R clean reads |
|---|---|---|---|---|
| Mean read length | 288.7 | 226.6 | 291.5 | 178.5 |
| Mean read quality | 22.3 | 29.6 | 17.6 | 27.7 |
| Number of reads | 1105532 | 935589 | 1105532 | 935589 |
| Total bases | 319153383 | 212010680 | 322259796 | 167040671 |

**Supplementary Table 4.** Statistics for Nanopore reads used for the assembly of the *K. humilis* MAW1 genome, before and after filtering.

| Statistics | Raw reads | Clean reads |
|---|---|---|
| Mean read length | 7823.1 | 8188.1 |
| Mean read quality | 10.8 | 11.6 |
| Number of reads | 392690 | 291705 |
| Total bases | 3072061280 | 2388514783 |
| N50 | 9276 | 9480 |
| Number, percentage, and bases of reads above quality cutoffs | | |
| Q5<br>Q7<br>Q10<br>Q12<br>Q15 | 390991 (99.6) 3071.0<br>377652 (96.2) 3004.0<br>292260 (74.4) 2388.8<br>106760 (27.2) 887.6<br>0 (0.0) 0.0 | 291705 (100.0) 2388.5<br>291705 (100.0) 2388.5<br>291705 (100.0) 2388.5<br>106621 (36.6) 887.6<br>0 (0.0%) 0.0Mb |
| The top three longest reads and their mean base quality | | |
| 1:<br>2:<br>3: | 172574 (6.4)<br>102396 (6.3)<br>94368 (11.7) | 94368 (11.7)<br>86916 (12.1)<br>86408 (11.6) |

**References**


[1] C. UniProt, UniProt: the universal protein knowledgebase in 2021, Nucleic Acids Res, 49 (2021) D480-D489.
[2] J.L. Gordon, D. Armisén, E. Proux-Wéra, S.S. ÓhÉigeartaigh, K.P. Byrne, K.H. Wolfe, Evolutionary erosion of yeast sex chromosomes by mating-type switching accidents, Proceedings of the National Academy of Sciences of the United States of America, 108 (2011) 20024-20029.
[3] G. Liti, A.N. Nguyen Ba, M. Blythe, C.A. Müller, A. Bergström, F.A. Cubillos, F. Dafhnis-Calas, S. Khoshraftar, S. Malla, N. Mehta, C.C. Siow, J. Warringer, A.M. Moses, E.J. Louis, C.A. Nieduszynski, High quality de novo sequencing and assembly of the Saccharomyces arboricolus genome, BMC Genomics, 14 (2013) 69.
[4] M.J. Stark, A.J. Mileham, M.A. Romanos, A. Boyd, Nucleotide sequence and transcription analysis of a linear DNA plasmid associated with the killer character of the yeast Kluyveromyces lactis, Nucleic Acids Res, 12 (1984) 6011-6030.
[5] D. Marquina, A. Santos, J. Peinado, Biology of killer yeasts, International Microbiology, 5 (2002) 65-71.
[6] R. Klassen, F. Meinhardt, Induction of DNA damage and apoptosis in Saccharomyces cerevisiae by a yeast killer toxin, Cellular Microbiology, 7 (2005) 393-401.
[7] T.W. Young, M. Yagiu, A comparison of the killer character in different yeasts and its classification, Antonie Van Leeuwenhoek, 44 (1978) 59-77.
[8] K.A. Bostian, Q. Elliott, H. Bussey, V. Burn, A. Smith, D.J. Tipper, Sequence of the preprotoxin dsRNA gene of type I killer yeast: multiple processing events produce a two-component toxin, Cell, 36 (1984) 741-751.
[9] N. Skipper, D.Y. Thomas, P.C. Lau, Cloning and sequencing of the preprotoxin-coding region of the yeast M1 double-stranded RNA, Embo j, 3 (1984) 107-111.
[10] E. Servienė, J. Lukša, I. Orentaitė, D.L.J. Lafontaine, J. Urbonavičius, Screening the Budding Yeast Genome Reveals Unique Factors Affecting K2 Toxin Susceptibility, PLOS ONE, 7 (2012) e50779.
[11] D. Dignard, M. Whiteway, D. Germain, D. Tessier, D.Y. Thomas, Expression in yeast of a cDNA copy of the K2 killer toxin gene, Mol Gen Genet, 227 (1991) 127-136.

[12] K. Goto, Y. Iwatuki, K. Kitano, T. Obata, S. Hara, Cloning and nucleotide sequence of the KHR killer gene of Saccharomyces cerevisiae, Agric Biol Chem, 54 (1990) 979-984.
[13] K. Goto, H. Fukuda, K. Kichise, K. Kitano, S. Hara, Cloning and nucleotide sequence of the KHS killer gene of Saccharomyces cerevisiae, Agric Biol Chem, 55 (1991) 1953-1958.
[14] F. IZGÜ, D. ALTINBAY, Isolation and Characterization of the K5-Type Yeast Killer Protein and Its Homology with an Exo-β-1,3-glucanase, Bioscience, Biotechnology, and Biochemistry, 68 (2004) 685-693.
[15] X. Wang, Z. Chi, L. Yue, J. Li, M. Li, L. Wu, A marine killer yeast against the pathogenic yeast strain in crab (Portunus trituberculatus) and an optimization of the toxin production, Microbiol Res, 162 (2007) 77-85.
[16] X. Wang, Z. Chi, L. Yue, J. Li, Purification and characterization of killer toxin from a marine yeast Pichia anomala YF07b against the pathogenic yeast in crab, Current microbiology, 55 (2007) 396-401.
[17] S. Muccilli, S. Wemhoff, C. Restuccia, F. Meinhardt, Exoglucanase-encoding genes from three Wickerhamomyces anomalus killer strains isolated from olive brine, Yeast, 30 (2013) 33-43.
[18] C. Kohchi, A. Toh-e, Nucleotide sequence of Candida pelliculosa beta-glucosidase gene, Nucleic Acids Res, 13 (1985) 6273-6282.
[19] F. Comitini, I. Mannazzu, M. Ciani, Tetrapisispora phaffii killer toxin is a highly specific β-glucanase that disrupts the integrity of the yeast cell wall, Microb Cell Fact, 8 (2009) 55.
[20] F. Comitini, N.D. Pietro, L. Zacchi, I. Mannazzu, M. Ciani, Kluyveromyces phaffii killer toxin active against wine spoilage yeasts: purification and characterization, Microbiology (Reading), 150 (2004) 2535-2541.
[21] M.J. Schmitt, D.J. Tipper, K28, a unique double-stranded RNA killer virus of Saccharomyces cerevisiae, Mol Cell Biol, 10 (1990) 4807-4815.
[22] J. Lu, B. Huang, A. Esberg, M.J. Johansson, A.S. Byström, The Kluyveromyces lactis gamma-toxin targets tRNA anticodons, Rna, 11 (2005) 1648-1654.
[23] N. Gunge, A. Tamaru, F. Ozawa, K. Sakaguchi, Isolation and characterization of linear deoxyribonucleic acid plasmids from Kluyveromyces lactis and the plasmid-associated killer character, Journal of bacteriology, 145 (1981) 382-390.
[24] M.J. Stark, Resolution of sequence discrepancies in the ORF1 region of the Kluyveromyces lactis plasmid k1, Nucleic Acids Res, 16 (1988) 771.
[25] R. Klassen, J.P. Paluszynski, S. Wemhoff, A. Pfeiffer, J. Fricke, F. Meinhardt, The primary target of the killer toxin from Pichia acaciae is tRNAGln, Molecular Microbiology, 69 (2008) 681-697.
[26] P.L. Worsham, P.L. Bolen, Killer toxin production in Pichia acaciae is associated with linear DNA plasmids, Curr Genet, 18 (1990) 77-80.
[27] A. Kast, R. Klassen, F. Meinhardt, rRNA fragmentation induced by a yeast killer toxin, Molecular Microbiology, 91 (2014) 606-617.
[28] G.T. Hayman, P.L. Bolen, Linear DNA plasmids of Pichia inositovora are associated with a novel killer toxin activity, Curr Genet, 19 (1991) 389-393.
[29] A. Allen, E. Islamovic, J. Kaur, S. Gold, D. Shah, T.J. Smith, Transgenic maize plants expressing the Totivirus antifungal protein, KP4, are highly resistant to corn smut, Plant Biotechnology Journal, 9 (2011) 857-864.
[30] C.M. Park, J.A. Bruenn, C. Ganesa, W.F. Flurkey, R.F. Bozarth, Y. Koltin, Structure and heterologous expression of the Ustilago maydis viral toxin KP4, Mol Microbiol, 11 (1994) 155-164.
[31] J. Tao, I. Ginsberg, N. Banerjee, W. Held, Y. Koltin, J.A. Bruenn, Ustilago maydis KP6 killer toxin: structure, expression in Saccharomyces cerevisiae, and relationship to other cellular toxins, Mol Cell Biol, 10 (1990) 1373-1381.
[32] C. Suzuki, S. Nikkuni, The primary and subunit structure of a novel type killer toxin produced by a halotolerant yeast, Pichia farinosa, J Biol Chem, 269 (1994) 3041-3046.
[33] C. Suzuki, S. Nikkuni, Purification and Properties of the Killer Toxin Produced by a Halotolerant Yeast, Pichia farinosa, Agricultural and Biological Chemistry, 53 (1989) 2599-2604.

[34] M.J. Schmitt, F. Neuhausen, Killer toxin-secreting double-stranded RNA mycoviruses in the yeasts Hanseniaspora uvarum and Zygosaccharomyces bailii, J Virol, 68 (1994) 1765-1772.
[35] F. Weiler, K. Rehfeldt, F. Bautz, M.J. Schmitt, The Zygosaccharomyces bailii antifungal virus toxin zygocin: cloning and expression in a heterologous fungal host, Mol Microbiol, 46 (2002) 1095-1105.
[36] S. Ashida, T. Shimazaki, K. Kitano, S. Hara, New Killer Toxin of Hansenula mrakii, Agricultural and Biological Chemistry, 47 (1983) 2953-2955.
[37] S. Kasahara, S. Ben Inoue, T. Mio, T. Yamada, T. Nakajima, E. Ichishima, Y. Furuichi, H. Yamada, Involvement of cell wall beta-glucan in the action of HM-1 killer toxin, FEBS Lett, 348 (1994) 27-32.
[38] T. Kimura, N. Kitamoto, K. Matsuoka, K. Nakamura, Y. Iimura, Y. Kito, Isolation and nucleotide sequences of the genes encoding killer toxins from Hansenula mrakii and H. saturnus, Gene, 137 (1993) 265-270.
[39] N. Rodríguez-Cousiño, M. Maqueda, J. Ambrona, E. Zamora, R. Esteban, M. Ramírez, A new wine Saccharomyces cerevisiae killer toxin (Klus), encoded by a double-stranded rna virus, with broad antifungal activity is evolutionarily related to a chromosomal host gene, Appl Environ Microbiol, 77 (2011) 1822-1832.
[40] F. Hishinuma, K. Nakamura, K. Hirai, R. Nishizawa, N. Gunge, T. Maeda, Cloning and nucleotide sequences of the linear DNA killer plasmids from yeast, Nucleic Acids Res, 12 (1984) 7581-7597.
[41] M. Tokunaga, A. Kawamura, F. Hishinuma, Expression of pGKL killer 28K subunit in Saccharomyces cerevisiae: identification of 28K subunit as a killer protein, Nucleic Acids Res, 17 (1989) 3435-3446.
[42] M. Tommasino, S. Ricci, C.L. Galeotti, Genome organization of the killer plasmid pGK12 from Kluyveromyces lactis, Nucleic Acids Res, 16 (1988) 5863-5878.
[43] F. Breinig, D.J. Tipper, M.J. Schmitt, Kre1p, the plasma membrane receptor for the yeast K1 viral toxin, Cell, 108 (2002) 395-405.
[44] F. Breinig, K. Schleinkofer, M.J. Schmitt, Yeast Kre1p is GPI-anchored and involved in both cell wall assembly and architecture, Microbiology (Reading), 150 (2004) 3209-3218.
[45] C. Boone, S.S. Sommer, A. Hensel, H. Bussey, Yeast KRE genes provide evidence for a pathway of cell wall beta-glucan assembly, J Cell Biol, 110 (1990) 1833-1843.
[46] M. Maftahi, J.M. Nicaud, H. Levesque, C. Gaillardin, Sequencing analysis of a 15.4 kb fragment of yeast chromosome XIV identifies the RPD3, PAS8 and KRE1 loci, five new open reading frames, Yeast, 11 (1995) 567-572.
[47] M. Maftahi, J.M. Nicaud, H. Levesque, C. Gaillardin, Sequencing analysis of a 24.7 kb fragment of yeast chromosome XIV identifies six known genes, a new member of the hexose transporter family and ten new open reading frames, Yeast, 11 (1995) 1077-1085.
[48] A. Häusler, P.W. Robbins, Glycosylation in Saccharomyces cerevisiae: cloning and characterization of an alpha-1,2-mannosyltransferase structural gene, Glycobiology, 2 (1992) 77-84.
[49] K. Hill, C. Boone, M. Goebl, R. Puccia, A.M. Sdicu, H. Bussey, Yeast KRE2 defines a new gene family encoding probable secretory proteins, and is required for the correct N-glycosylation of proteins, Genetics, 130 (1992) 273-283.
[50] R. Kölling, A. Lee, E.Y. Chen, D. Botstein, Nucleotide sequence of the SAC2 gene of Saccharomyces cerevisiae, Yeast, 10 (1994) 1211-1216.
[51] P. Meaden, K. Hill, J. Wagner, D. Slipetz, S.S. Sommer, H. Bussey, The yeast KRE5 gene encodes a probable endoplasmic reticulum protein required for (1----6)-beta-D-glucan synthesis and normal cell growth, Mol Cell Biol, 10 (1990) 3013-3019.
[52] N. Lertwattanasakul, T. Kosaka, A. Hosoyama, Y. Suzuki, N. Rodrussamee, M. Matsutani, M. Murata, N. Fujimoto, Suprayogi, K. Tsuchikane, S. Limtong, N. Fujita, M. Yamada, Genetic basis of the highly efficient yeast Kluyveromyces marxianus: complete genome sequence and transcriptome analyses, Biotechnol Biofuels, 8 (2015) 47.
[53] J.A. Varela, M. Puricelli, R.A. Ortiz-Merino, R. Giacomobono, S. Braun-Galleani, K.H. Wolfe, J.P. Morrissey, Origin of Lactose Fermentation in Kluyveromyces lactis by Interspecies Transfer of a Neo-functionalized Gene Cluster during Domestication, Curr Biol, 29 (2019) 4284-4290.e4282.

[54] A. Santos, M. San Mauro, C. Abrusci, D. Marquina, Cwp2p, the plasma membrane receptor for Pichia membranifaciens killer toxin, Mol Microbiol, 64 (2007) 831-843.
[55] Y. Hu, A. Rolfs, B. Bhullar, T.V. Murthy, C. Zhu, M.F. Berger, A.A. Camargo, F. Kelley, S. McCarron, D. Jepson, A. Richardson, J. Raphael, D. Moreira, E. Taycher, D. Zuo, S. Mohr, M.F. Kane, J. Williamson, A. Simpson, M.L. Bulyk, E. Harlow, G. Marsischky, R.D. Kolodner, J. LaBaer, Approaching a complete repository of sequence-verified protein-encoding clones for Saccharomyces cerevisiae, Genome Res, 17 (2007) 536-543.
[56] A.R. Butler, J.H. White, Y. Folawiyo, A. Edlin, D. Gardiner, M.J. Stark, Two Saccharomyces cerevisiae genes which control sensitivity to G1 arrest induced by Kluyveromyces lactis toxin, Mol Cell Biol, 14 (1994) 6306-6316.
[57] F. Frohloff, L. Fichtner, D. Jablonowski, K.D. Breunig, R. Schaffrath, Saccharomyces cerevisiae Elongator mutations confer resistance to the Kluyveromyces lactis zymocin, Embo j, 20 (2001) 1993-2003.
[58] L. Fichtner, F. Frohloff, K. Bürkner, M. Larsen, K.D. Breunig, R. Schaffrath, Molecular analysis of KTI12/TOT4, a Saccharomyces cerevisiae gene required for Kluyveromyces lactis zymocin action, Mol Microbiol, 43 (2002) 783-791.
[59] L. Fichtner, F. Frohloff, D. Jablonowski, M.J. Stark, R. Schaffrath, Protein interactions within Saccharomyces cerevisiae Elongator, a complex essential for Kluyveromyces lactis zymocicity, Mol Microbiol, 45 (2002) 817-826.
[60] D. Jablonowski, L. Fichtner, M.J. Stark, R. Schaffrath, The yeast elongator histone acetylase requires Sit4-dependent dephosphorylation for toxin-target capacity, Mol Biol Cell, 15 (2004) 1459-1469.
[61] F. Frohloff, D. Jablonowski, L. Fichtner, R. Schaffrath, Subunit communications crucial for the functional integrity of the yeast RNA polymerase II elongator (gamma-toxin target (TOT)) complex, J Biol Chem, 278 (2003) 956-961.
[62] B. Huang, M.J. Johansson, A.S. Byström, An early step in wobble uridine tRNA modification requires the Elongator complex, Rna, 11 (2005) 424-436.
[63] T.G. Petrakis, T.M. Søgaard, H. Erdjument-Bromage, P. Tempst, J.Q. Svejstrup, Physical and functional interaction between Elongator and the chromatin-associated Kti12 protein, J Biol Chem, 280 (2005) 19454-19460.
[64] C. Pallier, M. Valens, V. Puzos, H. Fukuhara, G. Chéret, F. Sor, M. Bolotin-Fukuhara, DNA sequence analysis of a 17 kb fragment of yeast chromosome XI physically localizes the MRB1 gene and reveals eight new open reading frames, including a homologue of the KIN1/KIN2 and SNF1 protein kinases, Yeast, 9 (1993) 1149-1155.
[65] G. Chéret, C. Pallier, M. Valens, B. Diagnan-Fornier, H. Fukuhara, M. Bolotin-Fukuhara, F. Sor, The DNA sequence analysis of the HAP4-LAP4 region on chromosome XI of Saccharomyces cerevisiae suggests the presence of a second aspartate aminotransferase gene in yeast, Yeast, 9 (1993) 1259-1265.
[66] A. Jacquier, P. Legrain, B. Dujon, Sequence of a 10.7 kb segment of yeast chromosome XI identifies the APN1 and the BAF1 loci and reveals one tRNA gene and several new open reading frames including homologs to RAD2 and kinases, Yeast, 8 (1992) 121-132.
[67] J.F. Diffley, B. Stillman, Similarity between the transcriptional silencer binding proteins ABF1 and RAP1, Science, 246 (1989) 1034-1038.
[68] L. Latchinian-Sadek, D.Y. Thomas, Expression, purification, and characterization of the yeast KEX1 gene product, a polypeptide precursor processing carboxypeptidase, J Biol Chem, 268 (1993) 534-540.
[69] K. Eisfeld, F. Riffer, J. Mentges, M.J. Schmitt, Endocytotic uptake and retrograde transport of a virally encoded killer toxin in yeast, Mol Microbiol, 37 (2000) 926-940.
[70] F. Riffer, K. Eisfeld, F. Breinig, M.J. Schmitt, Mutational analysis of K28 preprotoxin processing in the yeast Saccharomyces cerevisiae, Microbiology (Reading), 148 (2002) 1317-1328.

[71] A. Dmochowska, D. Dignard, D. Henning, D.Y. Thomas, H. Bussey, Yeast KEX1 gene encodes a putative protease with a carboxypeptidase B-like function involved in killer toxin and alpha-factor precursor processing, Cell, 50 (1987) 573-584.
[72] H. Zhu, H. Bussey, D.Y. Thomas, J. Gagnon, A.W. Bell, Determination of the carboxyl termini of the alpha and beta subunits of yeast K1 killer toxin. Requirement of a carboxypeptidase B-like activity for maturation, J Biol Chem, 262 (1987) 10728-10732.
[73] J.C. Wagner, D.H. Wolf, Hormone (pheromone) processing enzymes in yeast. The carboxy-terminal processing enzyme of the mating pheromone alpha-factor, carboxypeptidase ysc alpha, is absent in alpha-factor maturation-defective kex1 mutant cells, FEBS Lett, 221 (1987) 423-426.
[74] R.B. Wickner, Chromosomal and nonchromosomal mutations affecting the "killer character" of Saccharomyces cerevisiae, Genetics, 76 (1974) 423-432.
[75] R.B. Wickner, M.J. Leibowitz, Two chromosomal genes required for killing expression in killer strains of Saccharomyces cerevisiae, Genetics, 82 (1976) 429-442.
[76] K. Mizuno, T. Nakamura, T. Ohshima, S. Tanaka, H. Matsuo, Yeast KEX2 genes encodes an endopeptidase homologous to subtilisin-like serine proteases, Biochem Biophys Res Commun, 156 (1988) 246-254.
[77] R.S. Fuller, A. Brake, J. Thorner, Yeast prohormone processing enzyme (KEX2 gene product) is a Ca2+-dependent serine protease, Proceedings of the National Academy of Sciences of the United States of America, 86 (1989) 1434-1438.
[78] D. Pandolfo, A. De Antoni, G. Lanfranchi, G. Valle, The DNA sequence of cosmid 14-5 from chromosome XIV reveals 21 open reading frames including a novel gene encoding a globin-like domain, Yeast, 12 (1996) 1071-1076.
[79] C. Tanguy-Rougeau, M. Wésolowski-Louvel, H. Fukuhara, The Kluyveromyces lactis KEX1 gene encodes a subtilisin-type serine proteinase, FEBS Lett, 234 (1988) 464-470.
[80] C.B. Sharma, R. Knauer, L. Lehle, Biosynthesis of lipid-linked oligosaccharides in yeast: the ALG3 gene encodes the Dol-P-Man:Man5GlcNAc2-PP-Dol mannosyltransferase, Biol Chem, 382 (2001) 321-328.
[81] T. Kimura, N. Kitamoto, Y. Kito, Y. Iimura, T. Shirai, T. Komiyama, Y. Furuichi, K. Sakka, K. Ohmiya, A novel yeast gene, RHK1, is involved in the synthesis of the cell wall receptor for the HM-1 killer toxin that inhibits beta-1,3-glucan synthesis, Mol Gen Genet, 254 (1997) 139-147.
[82] L. Van Aelst, S. Hohmann, F.K. Zimmermann, A.W. Jans, J.M. Thevelein, A yeast homologue of the bovine lens fibre MIP gene family complements the growth defect of a Saccharomyces cerevisiae mutant on fermentable sugars but not its defect in glucose-induced RAS-mediated cAMP signalling, Embo j, 10 (1991) 2095-2104.
[83] B. Becker, A. Blum, E. Gießelmann, J. Dausend, D. Rammo, N.C. Müller, E. Tschacksch, M. Steimer, J. Spindler, U. Becherer, J. Rettig, F. Breinig, M.J. Schmitt, H/KDEL receptors mediate host cell intoxication by a viral A/B toxin in yeast, Sci Rep, 6 (2016) 31105.
[84] P. De Wergifosse, B. Jacques, J.L. Jonniaux, B. Purnelle, J. Skala, A. Goffeau, The sequence of a 22.4 kb DNA fragment from the left arm of yeast chromosome II reveals homologues to bacterial proline synthetase and murine alpha-adaptin, as well as a new permease and a DNA-binding protein, Yeast, 10 (1994) 1489-1496.
[85] M.J. Lewis, D.J. Sweet, H.R. Pelham, The ERD2 gene determines the specificity of the luminal ER protein retention system, Cell, 61 (1990) 1359-1363.
[86] J.C. Semenza, K.G. Hardwick, N. Dean, H.R. Pelham, ERD2, a yeast gene required for the receptor-mediated retrieval of luminal ER proteins from the secretory pathway, Cell, 61 (1990) 1349-1357.
