# Supplementary figures and images for: Unlocking the genome of the non-sourdough Kazachstania humilis MAW1: insights into inhibitory factors and phenotypic properties
Source: Microb Cell Fact. 2024 Apr 15;23:111. doi: 10.1186/s12934-024-02380-7 (PMC11017505; doi:10.1186/s12934-024-02380-7)

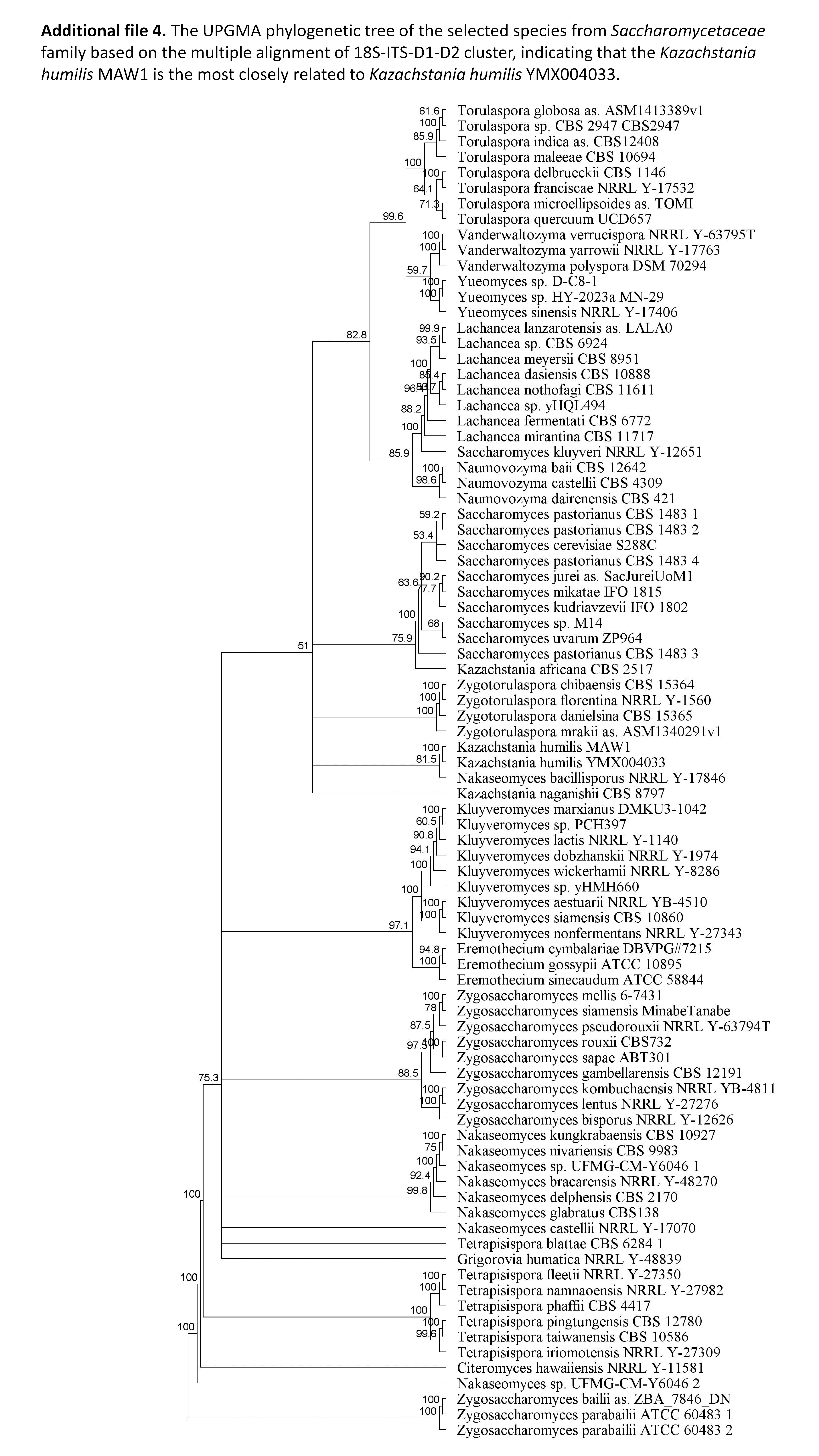

Supplement: Supplementary file 4 — Supplementary Material 4 [file 12934_2024_2380_MOESM4_ESM.jpg]
